# *Prevotella copri* increases fat accumulation in pigs fed with formula diets

**DOI:** 10.1186/s40168-021-01110-0

**Published:** 2021-08-21

**Authors:** Congying Chen, Shaoming Fang, Hong Wei, Maozhang He, Hao Fu, Xinwei Xiong, Yunyan Zhou, Jinyuan Wu, Jun Gao, Hui Yang, Lusheng Huang

**Affiliations:** 1 State Key Laboratory of Pig Genetic Improvement and Production Technology, Jiangxi Agricultural University, Nanchang, 330045 People’s Republic of China; 2 State Key Laboratory of Agricultural Microbiology, College of Animal Sciences and Technology, Huazhong Agricultural University, Wuhan, 430070 China

**Keywords:** Pigs; *P. copri*, Fat accumulation, Serum metabolites, Gavage experiment, Transcriptome

## Abstract

**Background:**

Excessive fat accumulation of pigs is undesirable, as it severely affects economic returns in the modern pig industry. Studies in humans and mice have examined the role of the gut microbiome in host energy metabolism. Commercial Duroc pigs are often fed formula diets with high energy and protein contents. Whether and how the gut microbiome under this type of diet regulates swine fat accumulation is largely unknown.

**Results:**

In the present study, we systematically investigated the correlation of gut microbiome with pig lean meat percentage (LMP) in 698 commercial Duroc pigs and found that *Prevotella copri* was significantly associated with fat accumulation of pigs. Fat pigs had significantly higher abundance of *P. copri* in the gut. High abundance of *P. copri* was correlated with increased concentrations of serum metabolites associated with obesity, e.g., lipopolysaccharides, branched chain amino acids, aromatic amino acids, and the metabolites of arachidonic acid. Host intestinal barrier permeability and chronic inflammation response were increased. A gavage experiment using germ-free mice confirmed that the *P. copri* isolated from experimental pigs was a causal species increasing host fat accumulation and altering serum metabolites. Colon, adipose tissue, and muscle transcriptomes in *P. copri*-gavaged mice indicated that *P. copri* colonization activated host chronic inflammatory responses through the TLR4 and mTOR signaling pathways and significantly upregulated the expression of the genes related to lipogenesis and fat accumulation, but attenuated the genes associated with lipolysis, lipid transport, and muscle growth.

**Conclusions:**

Taken together, the results proposed that *P. copri* in the gut microbial communities of pigs fed with commercial formula diets activates host chronic inflammatory responses by the metabolites through the TLR4 and mTOR signaling pathways, and increases host fat deposition significantly. The results provide fundamental knowledge for reducing fat accumulation in pigs through regulating the gut microbial composition.

Video Abstract

**Supplementary Information:**

The online version contains supplementary material available at 10.1186/s40168-021-01110-0.

## Background

Fatness traits such as backfat thickness and lean meat percentage (LMP) are economically important traits that affect the efficiency and return of modern pig production. Many factors can influence fat accumulation or fatness of pigs, including genetics, feed, and management. In humans and mice, an increasing number of studies using metagenomic sequencing analysis, colonization experiments of germ-free mice, or cohousing of mice harboring different gut microbiota have demonstrated that the gut microbiota is an important factor contributing to host obesity [1–5]. The gut microbiome of obese individuals often has a lower ratio of Bacteroidetes to Firmicutes and an increased capacity to harvest energy from the diet [5, 6]. The domestic pig is an ideal animal model for studying the causal role of gut microbiota in host obesity and fat accumulation because pigs are prone to deposit excess fat, and the diets of pigs are easily controlled. Furthermore, pigs show a high similarity of gastrointestinal structure with humans [7].

Despite a decade of research establishing a strong association between the gut microbiota and obesity, conflicting findings provided by several studies have challenged this view [8, 9]. There are few bacterial strains that have been isolated and confirmed as having causal roles in obesity [10–12]. Moreover, the underlying mechanism has not yet been clearly established [13], although several studies have indicated that obesity is associated with a low-grade systemic and chronic inflammatory condition [14, 15]. In the modern pig industry, to obtain rapid body weight gain, commercial formula diets with high concentrations of proteins and energy have often been provided to production pig herds. Whether and how the gut microbiome regulates swine fat accumulation (e.g., LMP) is also largely unknown.

In the current study, we investigated the relationship of gut microbial species with fat accumulation of pigs by performing an association study in 698 commercial Duroc pigs fed commercial diets (corn-soybean formula feeds containing 2960–3023 kcal/kg of digestible energy and 15–17% of protein). We identified *P. copri* as a main bacterial species increasing host fat accumulation (decreasing LMP) in pigs. High abundance of gut *P. copri* increased serum levels of lipopolysaccharide (LPS), branch chain amino acids (BCAAs), aromatic amino acids (AAAs), and the metabolites of arachidonic acid metabolism, thereby increasing host intestinal barrier permeability and causing a chronic inflammation response through the TLR4 and mTOR signaling pathways. The expression levels of genes related to lipid metabolism, transport, and localization in adipose and muscle tissues were significantly altered. To further confirm the causality of *P. copri* in host fat accumulation and investigate the diet effect on the colonization of *P. copri* isolated in this study, a gavage experiment using *P. copri* was carried out in germ-free mice (**Additional file** **1****: Fig. S1)**.

## Results

### Identifying a significant association of *P. copri* with fat accumulation of pigs

**Fig. 1:**
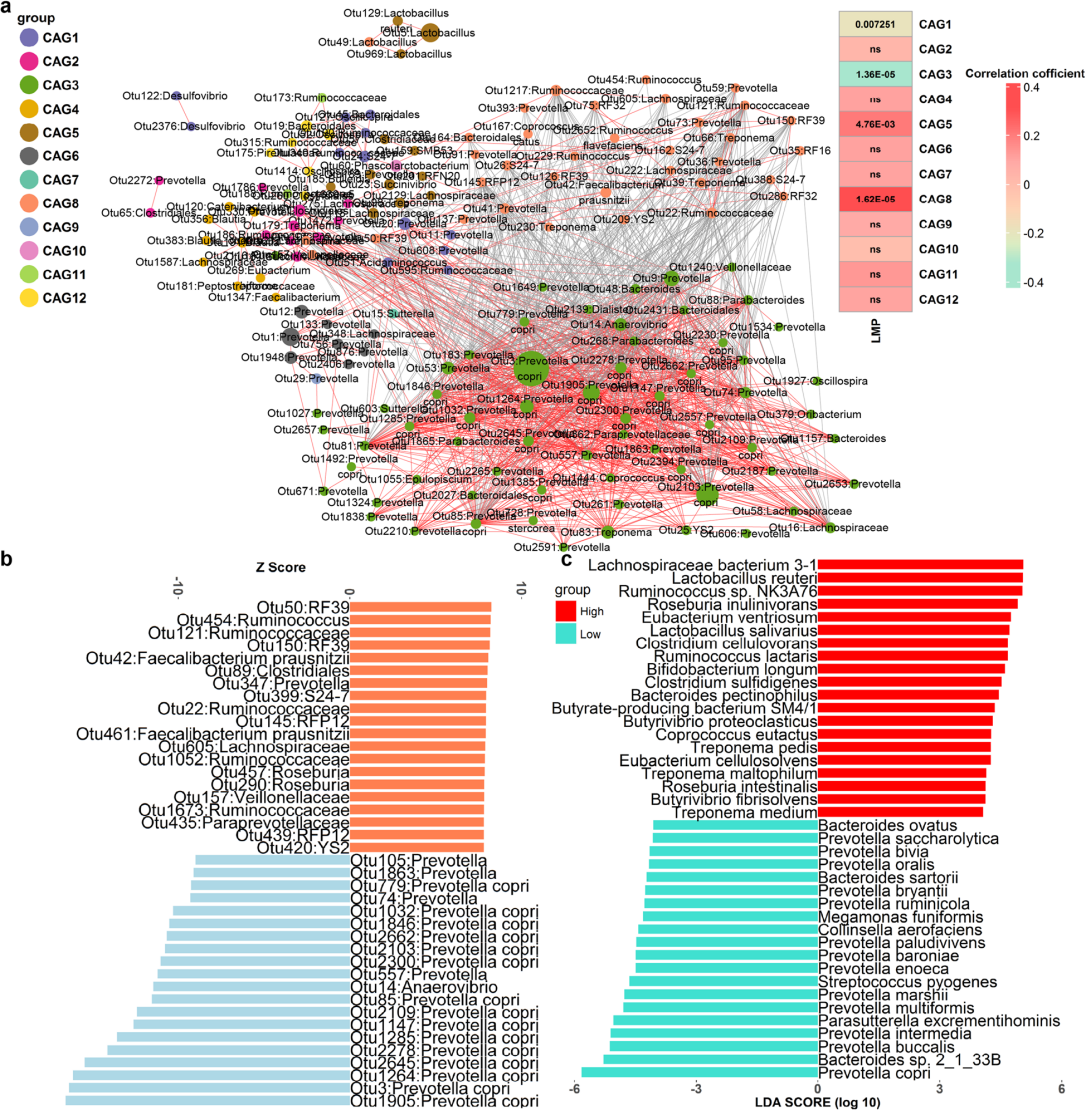
*P. copri* identified as a hub bacterial species decreasing lean meat percentage (LMP) of pigs. **a** OTU-level network diagram for identifying co-abundance groups (CAGs) responding to porcine LMP. Node sizes show the average abundance of each OTU. Red and grey lines between nodes indicate the positive and negative correlations between the nodes, respectively. Only lines corresponding to the correlations with a magnitude greater than 0.5 are drawn. The OTUs passing quality control (relative abundance ≥ 0.1% and present in at least 1% of pigs in the test cohort) were grouped into 12 CAGs by permutational multivariate analysis of variance (PERMANOVA) when *P* < 0.005. The color gradients on the right show the *P* values and coefficients (in the brackets) of the correlations between CAGs and the LMP. **b** Key OTUs of fecal microbiota associated with pig LMP. The histogram shows the *Z* scores computed for the OTUs significantly associated with the LMP using a two-part model. Only those OTUs for which the *Z* scores were ranked in the top 20 for positive (red bars) and negative (blue bars) association are shown. **c** Bacterial species significantly associated with the LMP using metagenomic sequencing data. The histogram shows the LDA scores computed for bacterial species differentially abundant between the samples with extremely high (*n* = 8) and low LMP (*n* = 8)

Excessive fat accumulation significantly decreases pig lean meat percentage (LMP). Therefore, in this study, we used the LMP as an index to assess the role of the gut microbiome in porcine fatness. We recorded the LMP of 698 commercial Duroc pigs raised in two farms. The discovery cohort comprised 550 pigs (309 males and 241 females) from two farms (280 from Shahu and 270 from Jiangying), and the validated cohort contained 148 pigs (100 males and 48 females) from the Jiangying farm (Methods). The phenotypic values generally fitted the normal distribution (**Additional file** **1****: Fig. S2**). All 698 pigs had fecal samples collected at the age of 160 days, and we performed hypervariable region sequencing of the 16S rRNA gene (V4 region for the discovery cohort and V3–V4 regions for the validation cohort). The descriptions of the sequencing procedures are summarized in **Additional file** **2****: Table S1**. We first analyzed the association of enterotypes and co-abundance groups (CAGs) of OTUs with the LMP in the discovery cohort. All samples were clustered into two enterotype-like groups that were dominated by either *Prevotella* or *Treponema*, and the pigs with the *Prevotella* enterotype had significantly lower LMP (**Additional file** **1****: Fig. S3**). At the CAG level, all 1,159 OTUs that passed quality control were used to construct a co-abundance network. These OTUs were clustered into 12 co-abundance groups (CAGs) based on SparCC correlation coefficients (Fig. 1a and **Additional file** **1****: Fig. S4**). The CAG3 containing the OTUs mostly annotated to *Prevotella*, especially *P. copri*, were negatively correlated with the LMP, while the CAG8 that contained the OTUs annotated to *F. prausnitzii* and *R. flavefaciens* showed strongly positive correlations with the LMP, suggesting the central roles of these CAGs in the functional guilds of gut microbiota related to the LMP (Fig. 1a).

We then performed a bacteria-wide association study with a two-part model to identify the bacterial taxa significantly associated with the LMP in the discovery cohort. A total of 166 LMP-associated OTUs were identified at FDR < 0.01, including 82 OTUs positively associated with the LMP and 84 OTUs showing negative associations with the LMP. Those positively associated OTUs mostly belonged to the order Clostridiales, for example, *F. prausnitzii*, Lachnospiraceae, and Ruminococcaceae, while the negatively associated OTUs were mainly aligned to *Prevotella* (40/84). In particular, 18 *P. copri* OTUs showed the strongest negative associations with the LMP (Fig. 1b and **Additional file** **2****: Table S2**).

The results of the enterotype analysis were well repeated in the validation cohort. The OTUs belonging to *Prevotella* (e.g., *P. copri*) were clustered into the CAG8 that was negatively associated with the LMP, and the OTU17 annotated to *P. copri* was the hub node in the co-abundance network (**Additional file** **1****: Fig. S5**). A total of 11 LMP-associated OTUs were identified in the validation cohort. Most of the bacterial taxa annotated to these LMP-associated OTUs were the same as those in the discovery cohort (8/11). Two out of the five OTUs positively associated with the LMP belonged to Christensenellaceae, while the OTUs showing the most significant negative association with the LMP were annotated to *Prevotella* and *P. copri* (**Additional file** **2****: Table S3**). These results further suggest the significant association of *P. copri* with fat accumulation of pigs.

We further performed shotgun metagenomic sequencing of 16 fecal samples that were contained in the validation cohort, including eight samples with the highest LMP values and another eight samples with the lowest LMP (**Additional file** **1****: Fig. S6a**). The metagenomic sequencing data are summarized in **Additional file** **2****: Table S4**. Consistent with the previous findings in humans [12, 16], the fat pigs had a significantly lower number of genes and α-diversity (Shannon index) in the gut microbiome than their lean counterparts (**Additional file** **1****: Fig. S6b, c**). We identified 40 species responsible for the LMP by a linear discriminant analysis of effect size (LEfSe). The members from *Prevotella* predominated the bacterial species enriched in fat pigs (13/20). In particular, *P*. *copri* showed the strongest negative association with the LMP and was the hub species among the bacterial species decreasing the LMP (**Additional file** **1****: Fig. S6d**). A total of 20 species were enriched in lean pigs, most of which were SCFAs-producing bacteria from *Treponema* and Clostridiales, e.g. *Lactobacillus reuteri* and *Bifidobacterium longum* (Fig. 1c). To extend the metagenomic sequencing data, we integrated the metagenomic sequencing data of 20 fecal samples from the discovery cohort that were chosen based on the phenotypic values of feed efficiency in our previous study [17]. Similar to the results in the 16 samples, the results in the integrated 36 samples showed that four species of *Prevotella* including *P. copri* were significantly associated with the decreased LMP, while the species from *Treponema* and Clostridiales had higher abundance in lean pigs (**Additional file** **1****: Fig. S7a**).

### Functional capacity of the gut microbiome related to fat accumulation of pigs

**Fig. 2:**
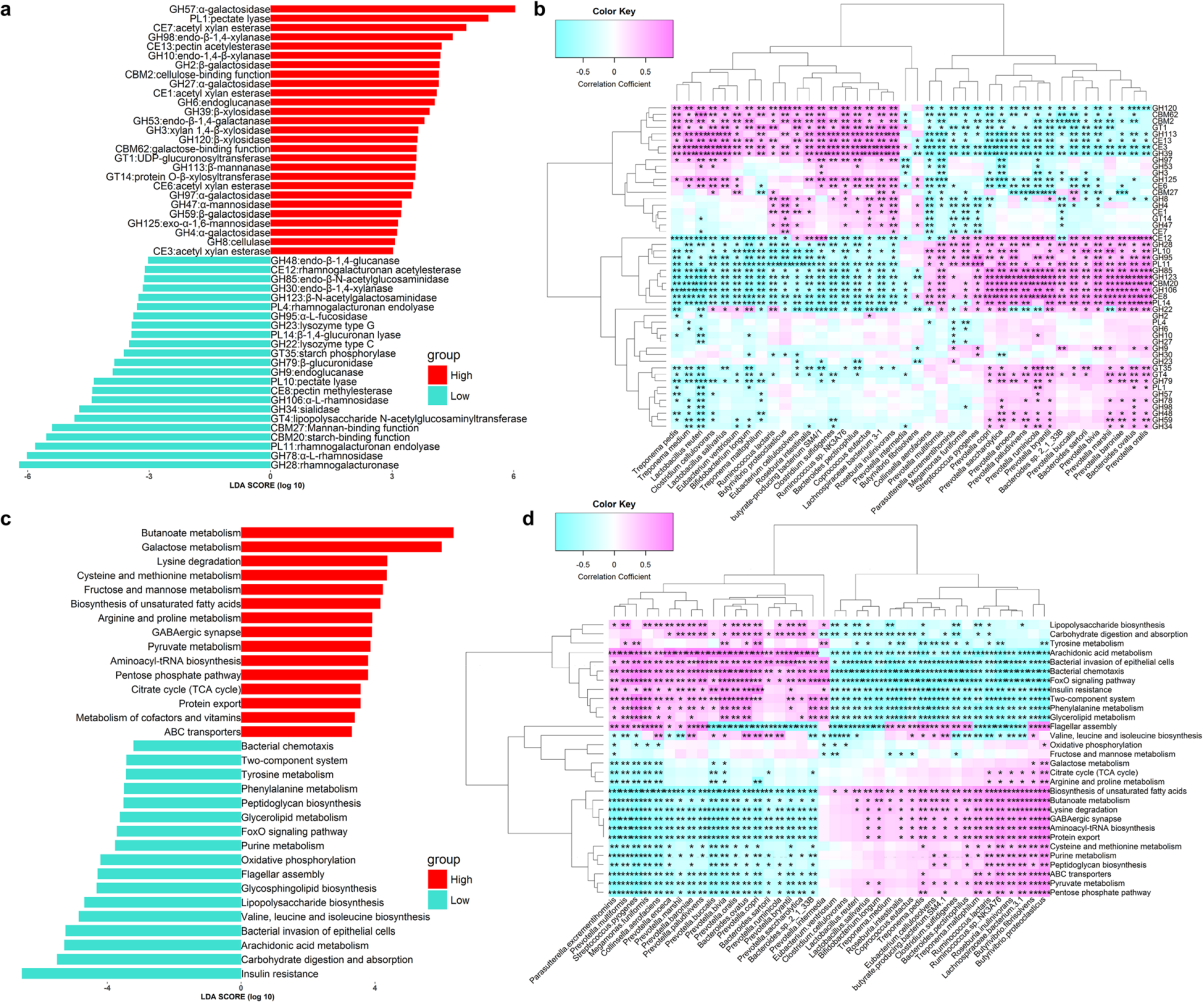
Functional capacities of the gut microbiome associated with pig lean meat percentage (LMP) and its correlation with the LMP-associated bacteria with available metagenomic data. **a** Function terms of CAZy associated with the LMP. **b** Correlations of the LMP-associated CAZymes with the LMP-associated bacterial species. **c** Functional terms of KEGG pathways associated with the LMP. **d** Correlations of the LMP-associated KEGG pathways with the LMP-associated bacterial species. The histogram shows the LDA scores computed for function terms differentially abundant between samples with high (*n* = 8) and low LMP (*n* = 8). The correlations between LMP-associated function terms and bacterial species were set at the threshold |*r*| > 0.5, FDR < 0.05

We next investigated the function capacities of the gut microbiome related to the LMP by mapping the microbial gene catalog onto the Carbohydrate-Active enZYmes database (CAZy), and Kyoto Encyclopedia of Genes and Genomes (KEGG) modules using metagenomic sequencing data. We identified a total of 50 CAZymes having significantly different abundances between fat and lean pigs, with 27 CAZymes involved in the metabolism of galactose, xylan, and mannose that were enriched in lean pigs. The 23 CAZymes having significantly higher abundance in the gut microbiome of obese individuals are mainly involved in the metabolism of rhamnose and glucan, and the biosynthesis of lipopolysaccharide (e.g., GH28, PL11, GH22, PL10 and GT4) (Fig. 2a). Correlation analysis between the LMP-associated OTUs and CAZymes indicated the contribution of the LMP-associated bacteria to the changes in CAZymes (Fig. 2b).

The LMP-associated KEGG pathways are shown in Fig. 2c and **Additional file** **1****: Fig. S7b**. We identified 17 KEGG pathways having significantly higher abundance in the gut microbiomes of fat pigs, including lipopolysaccharide biosynthesis and arachidonic acid metabolism involved in mediating inflammatory reactions [11, 18]; FoxO signaling pathway, insulin resistance, BCAA (valine, leucine, and isoleucine) biosynthesis, and metabolism of aromatic amino acids (tyrosine and phenylalanine, AAA) related to obesity and insulin resistance [12, 15], [19, 20] along with a two-component system, bacterial chemotaxis, flagellar assembly, and carbohydrate digestion and absorption associated with increased capacity for energy harvest from bacteria [6, 21]. The pathway bacterial invasion of epithelial cells that should impair gut barrier integrity was also highly enriched in fat pigs compared with lean pigs (Fig. 2c). All these pathways were positively correlated with the fatness-associated bacterial species, especially with *P. copri* (Fig. 2d), suggesting that the bacterial species from fat pigs may produce more factors related to inflammatory reactions, obesity and insulin resistance, impair host gut barrier integrity, and increase the capacity for energy harvesting.

**Fig. 3:**
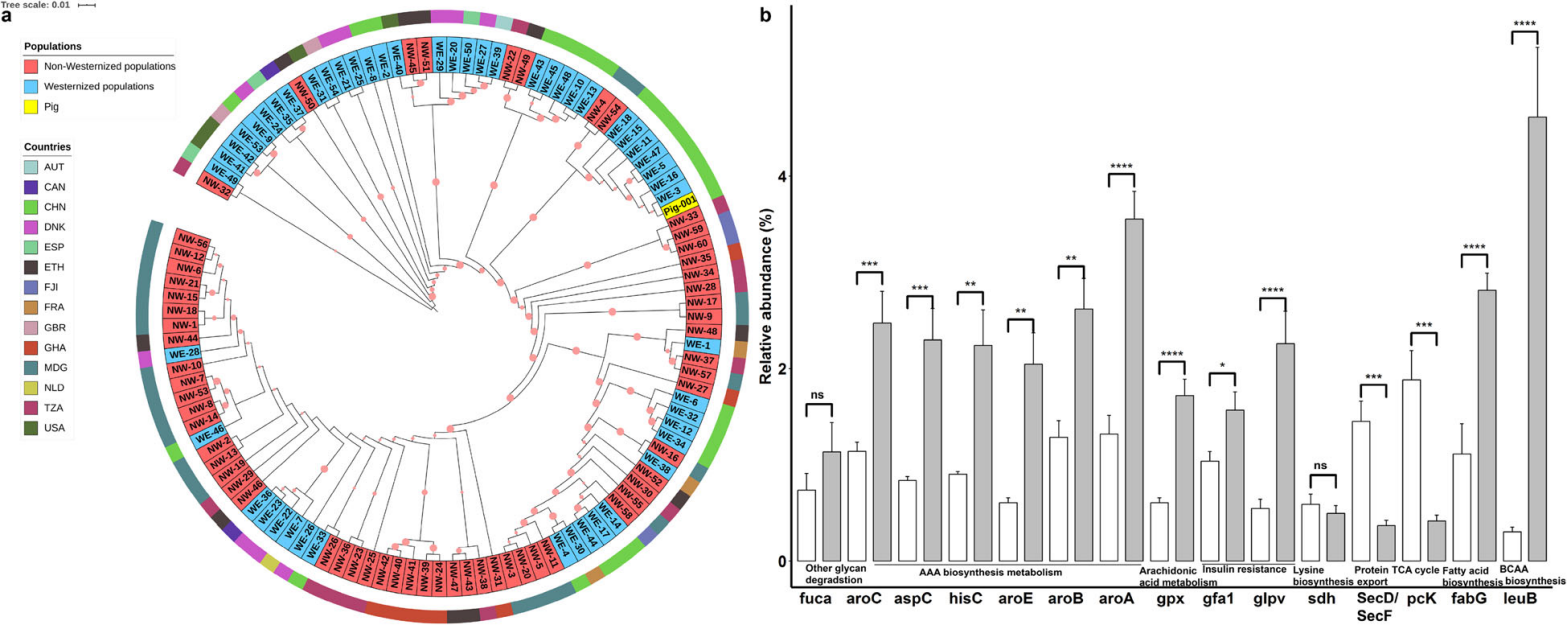
Genomic information of *P. copri* isolated in this study. **a** Phylogenetic tree of *P. copri* isolates from westernized and non-westernized human populations and from this study based on the genome sequences. Different colors of the outer circle represent the countries that samples were from; different colors of the inner circle indicate diets. **b** Comparison of abundances of the genes identified in the *P. copri* genome and involved in arachidonic acid metabolism, BCAA biosynthesis, AAA biosynthesis and metabolism, insulin resistance, the citrate cycle, and protein export between lean and fat pigs by integrating the metagenomic sequencing data; high: fat pigs; low: lean pigs. *FDR < 0.05, **FDR < 0.01, ***FDR < 0.005

We further isolated and cultured *P. copri* in vitro from the fecal samples of the experimental pigs with low LMP values. Whole-genome sequencing was performed using a Nanopore platform (“Methods”). The full-length of *P. copri* genome comprised 3.44 Mb containing 3,039 coding genes (CDS) (**Additional file** **1****: Fig. S8**). We first constructed a phylogenetic tree based on the genome sequences of *P. copri* isolates, including 60 isolates from westernized human populations, 51 isolates from non-westernized human populations, and one pig isolate from this study. The *P. copri* isolated from pigs in this study was clearly located in the clade from the westernized Chinese population (Fig. 3a). A total of 24 polysaccharide utilization loci (PULs) were then identified in this *P. copri* isolate. More than 10 PULs had higher prevalence in westernized populations (**Additional file** **1****: Fig. S9**). The genes involving in arachidonic acid metabolism, BCAA biosynthesis, AAA biosynthesis and metabolism, the TCA cycle, and protein export were found in the genome of this *P. copri* isolate, and the abundances of these genes in the tested samples were determined by combining the metagenomic sequencing data. Consistent with the LMP-associated functional capacities identified only by the metagenomic sequencing data, the gut microbiome of fat pigs had significantly higher abundances of the *P. copri* genes involved in arachidonic acid metabolism, BCAA biosynthesis, AAA biosynthesis and metabolism, and insulin resistance, but had lower abundances of the genes participating in the TCA cycle and protein export compared with lean pigs (Fig. 3b). Considering the high abundance of gut *P. copri* in fat pigs, *P. copri* was a main contributor to the shifts in metagenomic functional capacity related to the LMP.

Conversely, 15 KEGG pathways had significantly higher abundance in lean pigs (Fig. 2c), including butanoate metabolism, citrate cycle (the TCA cycle), metabolism of cofactors and vitamins, lysine degradation, cysteine and methionine metabolism, and arginine and proline metabolism. All these pathways were positively associated with multiple high LMP-associated bacterial species (Fig. 2d).

### The changes of serum metabolome in fat pigs and the correlation with shifts in gut microbiome

**Fig. 4:**
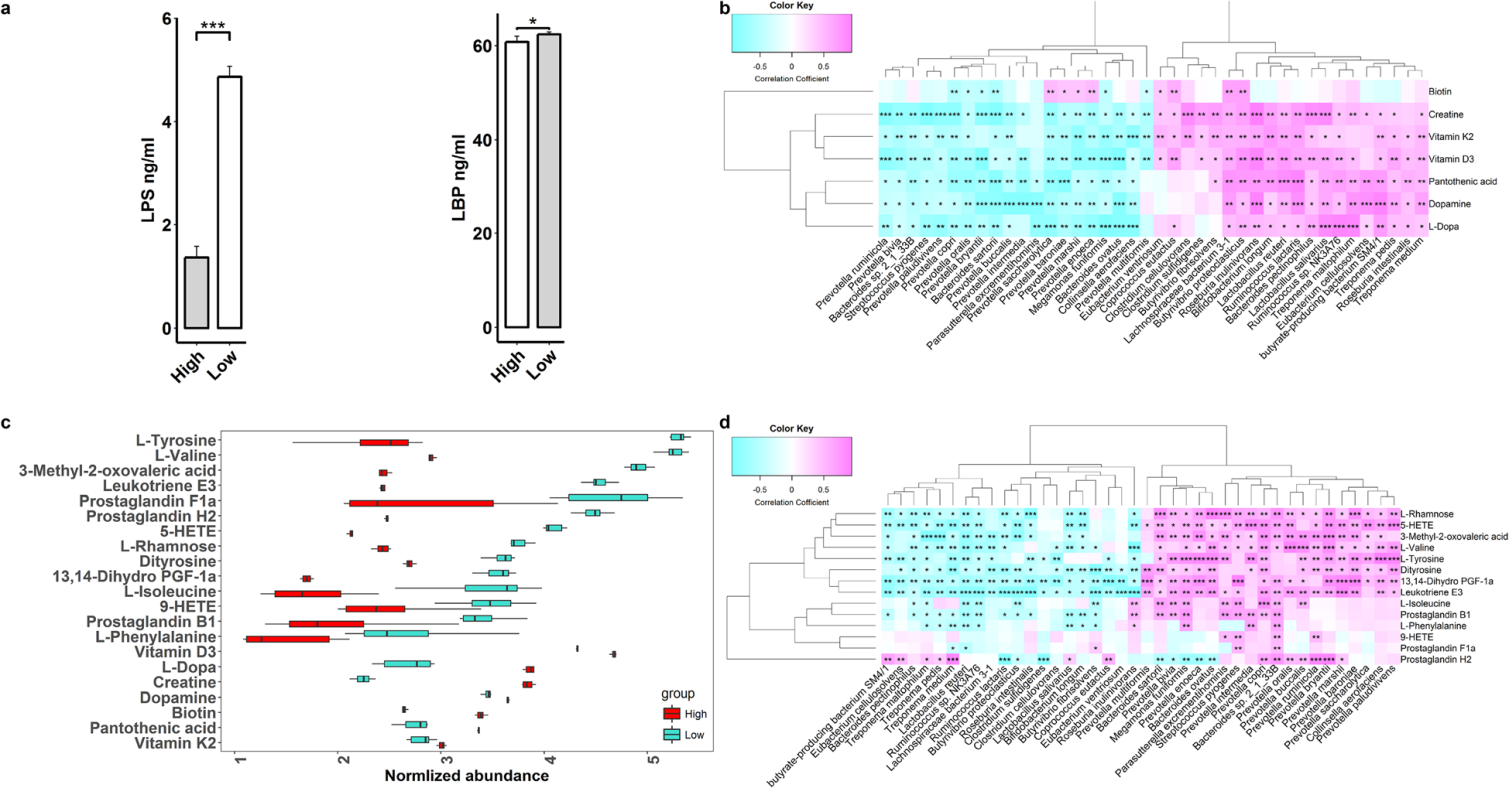
Lipopolysaccharide (LPS), lipopolysaccharide binding protein (LBP), and partial serum metabolites that differed in normalized abundance between fat and lean pigs, and their correlations with the LMP-associated bacterial species. **a** Comparison of the concentrations of serum LPS endotoxin and LBP between fat and lean pigs. High means the pigs with high LMP, and low represents the pigs with low LMP (fat pigs). **b** Metabolites that differed in normalized abundance between pigs with high (*n* = 8) and low LMP (*n* = 8). Red bars represent the normalized abundances of metabolites in lean pigs, blue bars show the normalized abundances of metabolites in fat pigs. **c** Correlations between the fatness-associated metabolites and the LMP-associated bacterial species. **d** Correlations between the high LMP-associated metabolites and the LMP-associated bacterial species. The color gradient represents the values of correlation coefficients

We first measured and compared the concentrations of serum LPS and lipopolysaccharide-binding protein (LBP) using an enzyme-linked immunosorbent assay (ELISA) between lean pigs (*n* = 8) and fat individuals (*n* = 8). Consistent with the higher abundance of the functional capacity of LPS biosynthesis in the gut microbiome, fat pigs had significantly higher serum LPS concentrations compared to their lean counterparts (*P* < 0.005). The same result was also observed for LBP (*P* < 0.05; Fig. 4a). We then determined non-targeted metabolome profiles of 38 serum samples randomly collected from the validated cohort. We identified 80 metabolite features showing significant association with the LMP by Spearman’s rank correlation analysis (FDR < 0.05) (**Additional file** **2****: Table S5**). These LMP-associated metabolites were clustered into 23 KEGG pathways covering most of the LMP-associated functional pathways of the gut microbiome (**Additional file** **1****: Fig. S10**). We next focused on some interesting LMP-associated metabolites based on the LMP-associated functional capacities of the gut microbiome. Serum concentrations of BCAA, AAA, and their related metabolites were considerably higher in fat pigs than in lean individuals (Fig. 4b and **Additional file** **1****: Table S5**). Furthermore, compared to their lean counterparts, fat pigs had significantly higher serum concentrations of the metabolites related to inflammatory reaction and metabolic syndrome, such as 3-methyl-2-oxovaleric acid, an intermediate of BCAA metabolism that can induce the accumulation of BCAAs [22], l-rhamnose (an important component of lipopolysaccharides), and the metabolites of arachidonic acid metabolism (5-HETE, 9-HETE, leukotrienes, and prostaglandins) (Fig. 4b and **Additional file** **2****: Table S5**). These were notably consistent with the proposed function capacity of the gut microbiome for BCAA biosynthesis and the metabolism of AAAs and arachidonic acid in fat pigs (Fig. 2b).

Compared to fat pigs, lean pigs had higher concentrations of the metabolites previously reported to reduce fat accumulation of pigs and increase lean muscle mass in serum, e.g., creatine [23] and the metabolites of betaine [24] (l-histidine trimethyl betaine and proline betaine) and anti-inflammatory factors (Lipoxin A4) [25] (Fig.4b and **Additional file** **2****: Table S5**). Catecholamines, including dopamine, N-acetyl dopamine, and l-dopa, which can reduce lipid accumulation in adipose tissue by increasing lipolysis, thereby decreasing lipogenesis and promoting free fatty acid (FFA) transportation [26], also exhibited higher abundances in the serum of lean pigs (**Additional file** **2****: Table S5 and** Fig. 4b). Serum concentrations of vitamins (vitamin K1, K2, D3, biotin, and pantothenic acid) were also significantly higher in lean pigs than in fat individuals (**Additional file** **2****: Table S5**).

We further evaluated the contribution of the LMP-associated bacteria to the shifts in serum metabolites at the OTU level in 38 samples with metabolome profiles (**Additional file** **1****: Fig. S11**) and at the species level in 16 samples having both metagenomic sequencing and metabolome data (Fig. 4c). *P. copri* was positively correlated with nearly all fatness-associated metabolites mentioned above, but negatively associated with the lean-associated metabolites (Fig. 4d). The other LMP-associated bacteria also contributed to the LMP-associated metabolites to different extents. For example, both *P. copri* and two *Bacteroides* spp. were significantly associated with serum BCAA concentration, but serum BCAA was largely driven by *P. copri* (**Additional file** **1****: Fig. S12**). These results indicated a significant contribution of the LMP-associated bacteria to the shifts in host serum metabolites related to fat accumulation.

Taken together, chronic inflammation-associated metabolites, e.g., BCAA, AAA, and metabolites of arachidonic acid, had higher abundances in fat pigs, while the metabolites associated with anti-inflammation, lipid metabolism, and energy expenditure were enriched in the serum of lean pigs. The *P. copri* and other LMP-associated bacteria responded to the shifts in serum metabolites in fat pigs.

### Increased host intestinal barrier permeability and chronic inflammatory reaction in fat pigs

**Fig. 5:**
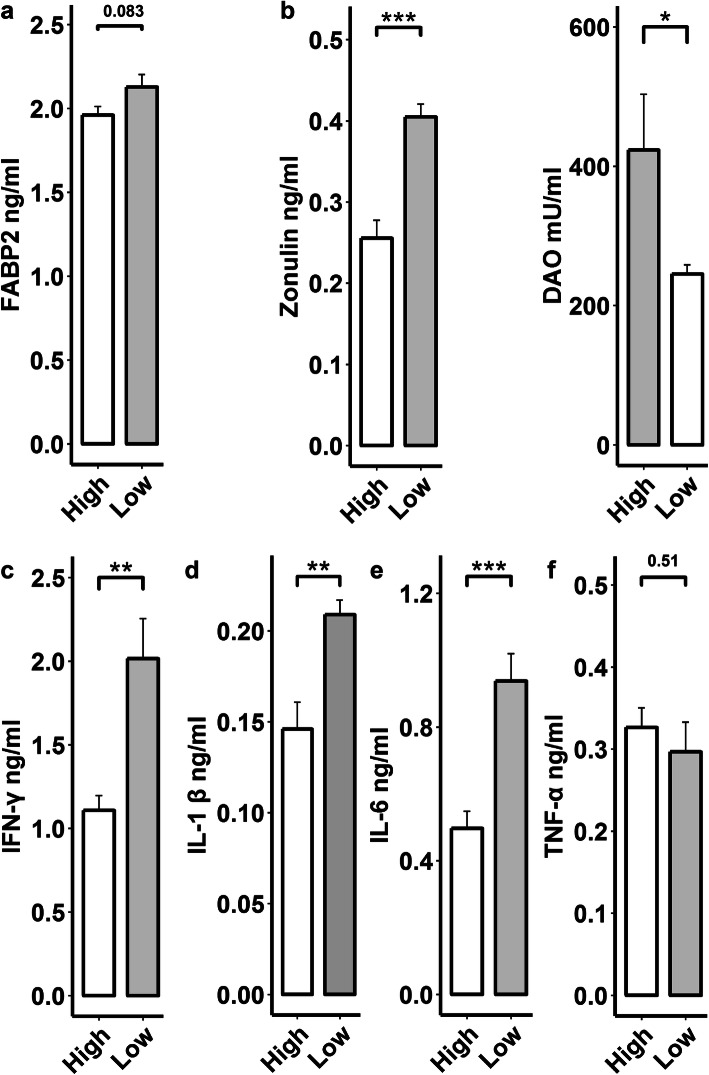
Comparison of serum concentrations of blood markers of gut permeability pathophysiological epithelium integrity (fatty acid-binding protein 2 (FABP2), zonulin and diamine oxidase (DAO)), and pro-inflammatory cytokines between the pigs with high (*n* = 8) and low (*n* = 8) lean meat percentage (LMP). **a**, **b** Comparison of serum concentrations of FABP2, zonulin and DAO between lean and fat pigs. **c**–**f** Comparison of serum concentrations of pro-inflammatory cytokines (IL-1β, IL-6, TNF-α, and IFN-γ) between lean and fat pigs. *P < 0.05; **P < 0.01, and ***P < 0.005. The results indicated that high abundance of gut *P. copri* upregulated gut barrier permeability and host pro-inflammatory reaction

Given the increased concentrations of serum LPS and the metabolites related to inflammatory reactions in fat pigs, in order to examine the host intestinal barrier permeability and chronic inflammation response, we determined the serum levels of biomarkers zonulin, diamine oxidase (DAO), and FABP2 [27] and the pro-inflammatory cytokines tumor necrosis factor α (TNF-α), interleukin-1β (IL-1β), interleukin-6 (IL-6), and interferon-γ (IFN-γ). As expected, compared to high lean meat pigs (*n* = 8), fat pigs (*n* = 8) had higher serum concentrations of zonulin (*P*< 0.005) and FABP2 (*P* = 0.083) although no significant difference was found in DAO (Fig. 5a), suggesting an increased intestinal barrier permeability in fat pigs. Moreover, fat pigs also had higher serum concentrations of TNF-α, IL-1β, IL-6, and IFN-γ (Fig. 5b), indicating host chronic inflammation response. Taken together, the results of metabolome analysis suggest that a high abundance of gut *P. copri* may induce host intestinal barrier permeability and promote chronic inflammatory response through the metabolites of LPS, BCAA, AAA, and arachidonic acid, and thereby increase fat accumulation in pigs.

### Gavage in germ-free mice confirmed the causal role of *P. copri*

**Fig. 6:**
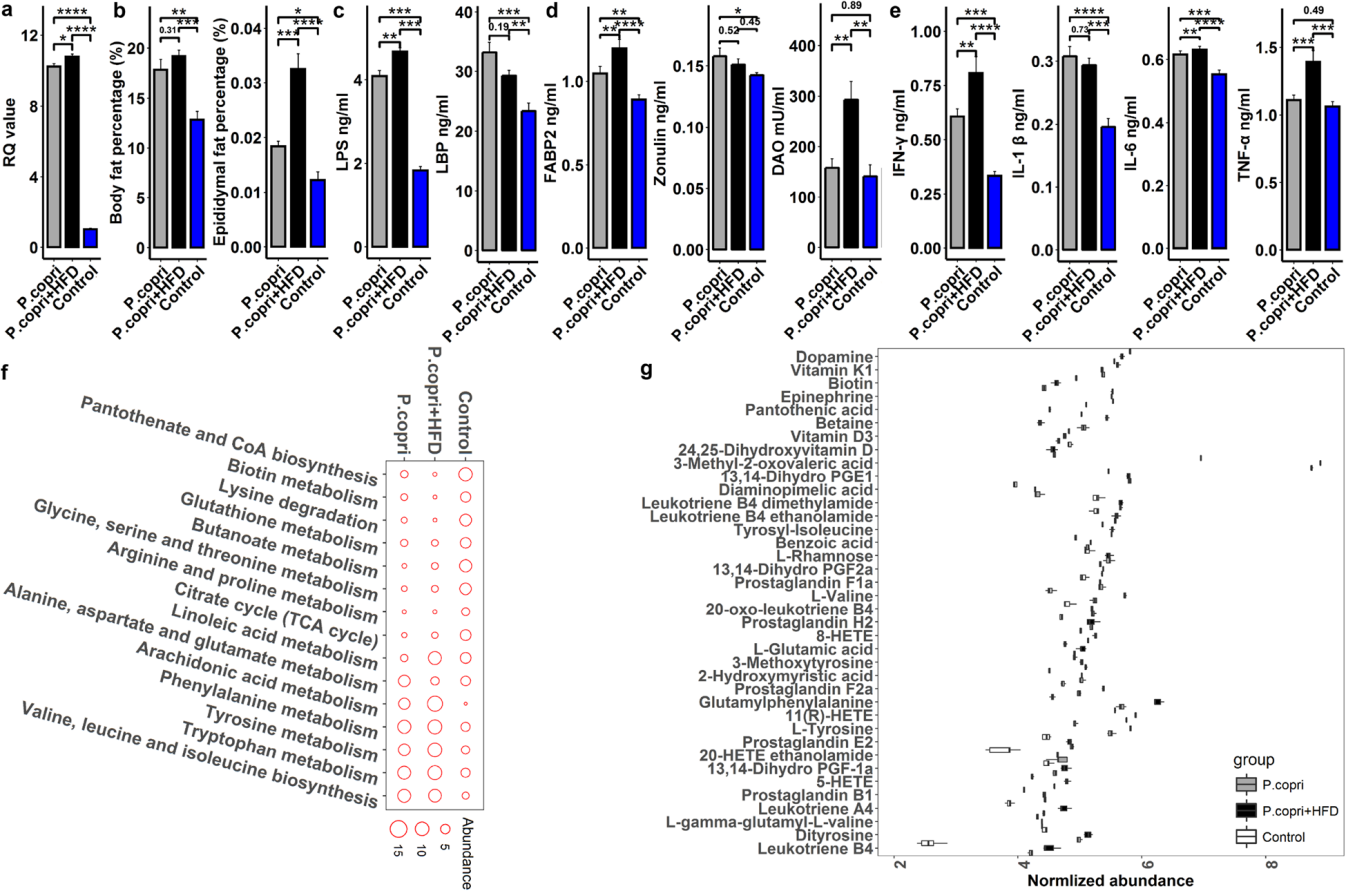
Confirmation of the causal role of *P. copri* in host fat accumulation. Live *P. copri* was administrated by oral gavage to germ-free mice three times per week at a dose of 100-μl bacterial suspension (1 × 10^7^ CFUs/μl). **a** qPCR confirmed the successful colonization of *P. copri* in germ-free mice. The *Y*-axis indicates the RQ values reflecting the relative abundance of *P. copri* in gavaged mice. **b** Comparison of body fat percentage (%) and epididymal fat percentage (%) among *P. copri*-gavaged mice (*n* = 7), *P. copri*-gavaged mice fed a high-fat diet (*n* =7), and control mice (*n* =7). Compared with control mice, *P. copri* colonization significantly increased host fat accumulation in both normal-chow mice and high-fat-feeding mice. **c** Comparison of serum concentrations of LPS endotoxin and LBP among experimental mouse groups. **d** Comparison of serum concentrations of FABP2, zonulin, and DAO among experimental mouse groups. **e** Comparison of serum concentrations of pro-inflammatory cytokines (IL-1β, IL-6, TNF-α, and IFN-γ) among experimental mouse groups. **f** Comparison of the KEGG pathways enriched by differential serum metabolites among experimental mouse groups. The red circles represent experimental mouse groups, and the sizes of red circles indicate the enrichment by differential metabolites. **g** Metabolites that differed in normalized abundance among experimental mouse groups. *P < 0.05, **P < 0.01, ***P < 0.005, all *P* values were adjusted for the multiple tests

We next evaluated the possible causal relationship between *P. copri* isolated from experimental pigs and host fat accumulation via a gavage experiment using live *P. copri* in germ-free mice. A qPCR analysis of fecal DNA at the end of a 1-month gavage experiment confirmed the successful colonization of *P. copri* in the guts of each treated mouse (Fig. 6a). Phenotype measurements found significantly increased body fat percentage (*P* < 0.01) and epididymis fat percentage (*P* < 0.05) in *P. copri*-gavaged mice raised on a normal chow diet (Fig. 6b). Fat accumulation became more severe in *P. copri*-gavaged mice fed a high-fat diet (HFD) (*P* < 0.005) (Fig. 6b).

Gavage with the bacteria significantly increased serum concentrations of both LPS and LBP (*P* < 0.005). Moreover, the serum concentration of LPS was enhanced by the HFD (Fig. 6c). *P. copri* colonization also caused increased serum concentrations of the intestinal barrier permeability biomarkers FABP2 (*P* < 0.01) and zonulin (*P* < 0.05). Notably, feeding a high-fat diet to colonized mice reinforced intestinal barrier permeability. The serum concentration of DAO increased near twofold in mice colonized by *P. copri* and fed a high-fat diet (Fig. 6d). Notably, serum concentrations of pro-inflammatory cytokines (IL-6, IL-1β, TNF-α, and IFN-γ) were significantly higher in *P. copri*-colonized mice than in PBS-gavaged control mice. This was enhanced by feeding a high-fat diet to *P. copri*-colonized mice (*P* < 0.01) (Fig. 6e).

We identified a total of 222 serum metabolites showing differential abundances between controls and *P. copri*-colonized mice and 215 differential metabolite features between controls and *P. copri*-colonized mice fed with HFD (**Additional file** **2****: Table S6**). The differential metabolites between controls and *P. copri*-colonized mice were enriched to the pathways highly similar to those identified between lean and fat pigs (**Additional file** **1****: Fig. S13**). For example, *P. copri* colonization (in both normal chow and HFD) increased the richness of the pathways related to BCAA biosynthesis, AAA metabolism, and arachidonic acid metabolism. The pathways of biotin metabolism, butanoate metabolism, and pantothenate were in low abundance in *P. copri*-colonized mice (Fig. 6f). Furthermore, as observed in pigs, the metabolites involved in inflammatory reactions and metabolic syndrome such as BCAA, AAA, leukotrienes, prostaglandins, HETE, and l-rhamnose had higher abundances in mice treated by gavage with *P. copri*, while the metabolites associated with lean muscle mass, energy expenditure and reduced lipid accumulation (e.g., betaine, vitamins, and dopamine) were lower in the serum of colonized mice (Fig. 6g and **Additional file** **2****: Table S6**).

### Transcriptome analysis of colon, adipose, and muscle tissues from *P. copri*-gavaged mice elucidated the mechanism of gut microbiome affecting host fat accumulation

**Fig. 7:**
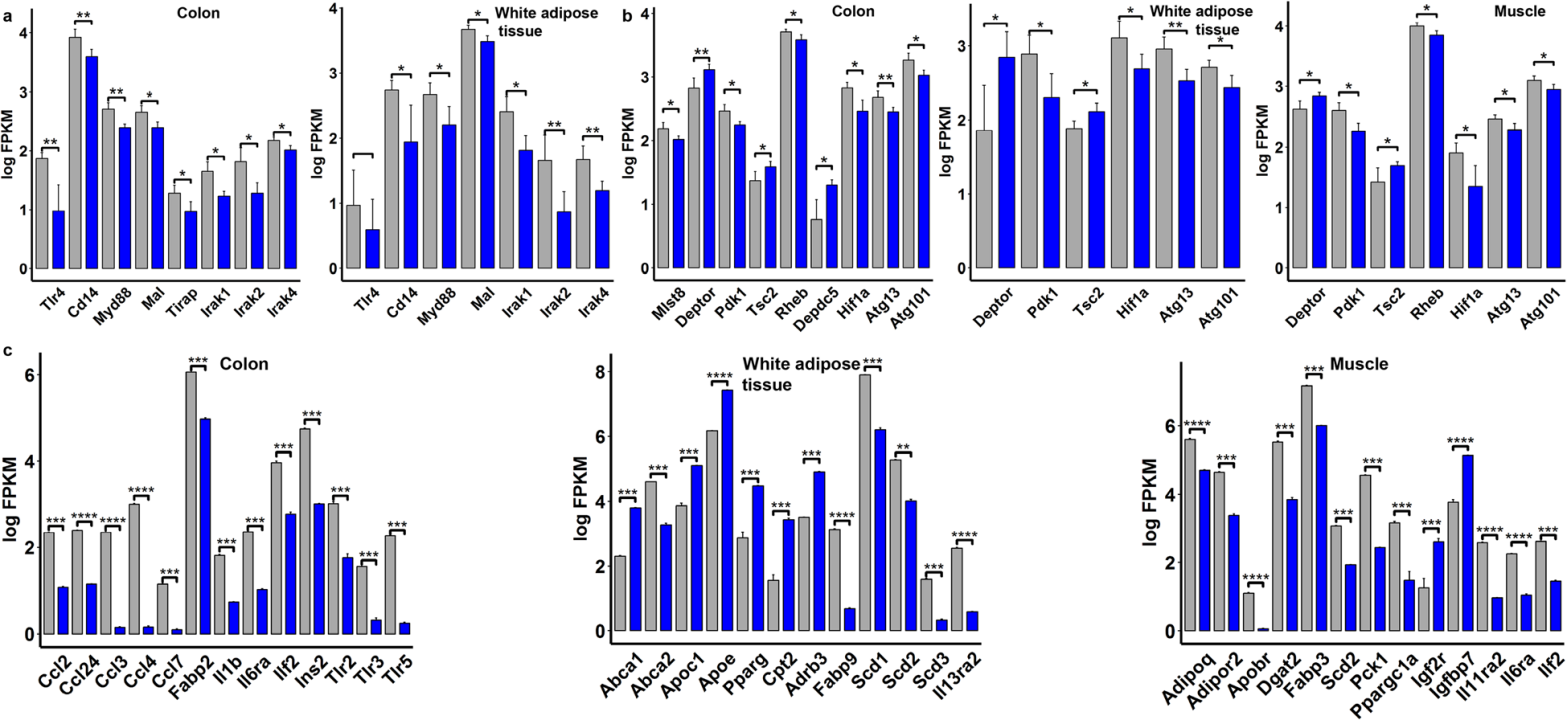
Changes in gene expression levels of host colon, adipose, and muscle tissues induced by *P. copri* administration. **a** The differential expression levels of the genes in TLR4 signaling cascades. **b** The expression changes of the genes in the mTOR pathway. **c** The differential expression levels of those genes involved in immunity, lipolysis, lipid transport, and muscle growth in the colon, white adipose tissue, and muscle between control and *P. copri*-colonized mice. *Corrected *P* < 0.05, **< 0.01, and ***< 0.005

To elucidate the molecular mechanism of *P. copri* influencing host body fat percentage, RNA-sequencing analysis was performed on the tissues of the colon, white adipose, and muscle harvested from control and colonized mice on a normal chow diet. There were 225, 338, and 384 differentially expressed genes (DEGs) identified between control and *P. copri*-colonized mice in colon, white adipose, and muscle tissues (FDR < 0.05), respectively (**Additional file** **2****: Table S7**). According to the increased concentrations of serum LPS, LBP, BCAA, and pro-inflammatory cytokines, we particularly focused on the expression of the genes in the TLR4 and mTOR signaling pathways that responded to bacterial LPS and BCAA, respectively [28]. As expected, compared with controls, mice colonized by *P. copri* had higher expression levels of *TLR4*, *CD14*, *Myd88*, *Mal*, *Irak1*, *Irak2*, and *Irak4* in both colon and epididymal fat tissues (FDR < 0.05), but not in muscle (Fig. 7a). This suggested the activation of the TLR4 signaling pathway in colon and white adipose tissues. The mTOR signaling pathway was also activated. *Mlst8*, a core component of mTOR Complex 1 (mTORC1) [29], was upregulated in colon tissues. *Rheb*, *Pdk1*, *Atg 13*, and *Atg101* were upregulated in all three tissues. However, *Tsc2* (a modulator of mTORC1) and *Deptor* (the inhibitory subunit of mTORC1) [30] were downregulated in all three tissues. Previous studies indicated that activation of mTORC1 resulting in lipid accumulation was tightly coupled to upregulation of *Hif1a* [31]. Interestingly, *Hif1a* was upregulated in all three tissues (Fig. 7b).

We also identified some interesting DEGs related to immune and inflammatory responses, and fat accumulation and obesity. In the colon tissue of *P. copri*-colonized mice, genes involved in immune and inflammatory responses (e.g., *Ccl2*, *Ccl24*, *Ccl3*, *Ccl4*, *Ccl7*, *Il1b*, *Il6ra*, *Ilf2*, *Tlr2*, *Tlr3*, *Tlr5*, and six genes from the immunoglobulin superfamily), and genes involved in fat accumulation and obesity (such as *Fabp2* and *Ins2*) had higher mRNA levels (Fig. 7c and **Additional file** **2****: Table S7**). This was consistent with the chronic inflammatory reaction and the increased intestinal permeability induced by *P. copri*. In white adipose tissue, DEGs involved in lipogenesis (*Fabp9*, *Scd1*, *Scd2*, and *Scd3*) and inflammatory response (*Il13ra2*) showed higher expression levels in *P. copri*-colonized mice than in control mice. However, several genes related to lipolysis and lipid transport (*Abca1*, *Apoc1*, *Apoe*, *Pparg*, *Cpt2*, and *Adrb3*) had lower expression levels in *P*. *copri*-colonized mice (Fig. 7c). In muscle, several genes related to lipogenesis and lipid deposition (e.g., *Adipoq*, *Adipor2*, *Apobr*, *Dgat2*, *Fabp3*, *Scd2*, *Pck1*, and *Ppargc1a*) and inflammatory response (*Il11ra2*, *Il6ra*, and *Ilf2*) had higher expression levels in *P. copri-*colonized mice, whereas *Igf2r* and *Igfbp7* associated with skeletal muscle growth were attenuated in response to *P. copri* colonization (Fig. 7c). Taken together, the results indicate that *P. copri* colonization activated the TLR4 and mTOR signaling pathways and upregulated the expression levels of the genes related to immune and inflammatory responses and the genes associated with lipogenesis and fat accumulation, while downregulating the expression levels of the genes associated with lipolysis, lipid transport, and muscle growth.

### The effect of diets on the colonization of *P. copri* strain isolated in this study

**Fig. 8:**
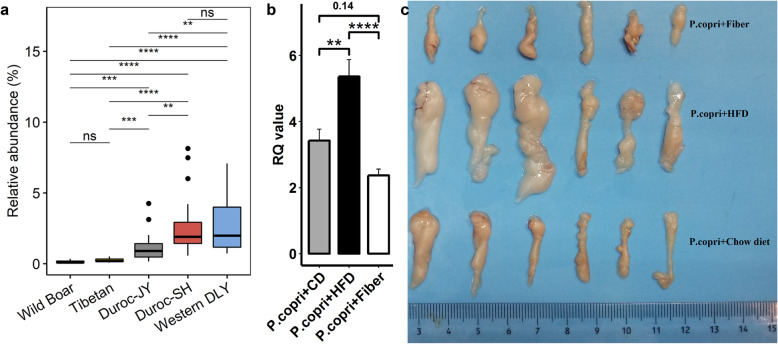
Diet effect on the abundance of gut *P. copri*. **a** Comparison of the abundances of *P. copri* in the guts of wild boars, Tibetan pigs, Duroc pigs, and Duroc × (Landrace × Yorkshire) pigs (DLY, *n* = 20) under industrial pig growing setting. **P < 0.01, ***P < 0.005, and ****P < 0.001. **b** Diet effect on fat accumulation in *P. copri*-gavaged mice. The C57BL6 germ-free mice colonized by *P. copri* were randomly divided into three groups fed with standard chow (CD, *n* = 6), a high-fat diet (HFD, *n* = 6), and a high-fiber diet (Fiber, *n* = 6). Significantly higher percentages (%) of body fat and epididymal fat were observed in the HFD group

Previous researches have shown that different habitual diets can influence the genomic function and strain representation of intestinal *P. copri* [32, 33]. We first carried out a comparison of the abundance of *P. copri* in the guts of pigs raised under different feeding patterns using metagenomic sequencing data from wild boars (*n* = 6; free-living, high-fiber diets), Tibetan pigs (*n* = 13; semi-grazing, high-fiber diets), Duroc pigs described above (*n* = 20 and 16; formula diets with high energy and protein), and Duroc × (Landrace × Yorkshire) pigs (DLY, *n* = 20) under industrial pig husbandry settings [34]. Significantly higher abundances of *P. copri* were observed in both Duroc populations (2.68% ± 0.49% (SE) and 1.20% ± 0.28%, percentage of reads mapped to *P. copri* of total clean reads) and DLY (2.67% ± 0.41%) compared to those in wild boars (0.15% ± 0.05%) and Tibetan pigs (0.23% ± 0.03%) (Fig. 8a, *P* < 0.005).

We further investigated the diet effect on *P. copri* colonization with C57BL6 germ-free mice. Eighteen germ-free mice were divided into three groups fed standard chow, a high-fat diet, or a high-fiber diet (“Methods”) and were given *P. copri* by gavage. A significantly higher abundance of colonized *P. copri* was identified in mice fed the high-fat diet (*P* < 0.01), but there was no significant difference between mice fed standard chow and those fed the high-fiber diet (Fig. 8b). Furthermore, compared to the mice fed standard chow, the mice fed a high-fat diet had significantly higher percentages of both body fat and epididymis fat (*P* < 0.005). The mice fed the high-fiber diet showed less fat accumulation than the mice fed standard chow (*P* < 0.05), but the difference was not large (Fig. 8c, **Additional file** **1****: Fig. 14**). This should be due to the diet provided to the high-fiber diet group, whose diet contained less carbohydrate and energy compared to the standard chow. As for the effects of diet on host intestinal barrier permeability and chronic inflammatory reaction in *P. copri-*colonized mice, there were no significant differences in the concentrations of LPS, LBP, biomarkers zonulin and FABP2, pro-inflammatory cytokines (IL-1β and IL-6), or TNF-ɑ between *P. copri-*gavaged mice fed standard chow and those fed a high-fiber diet (**Additional file** **1****: Fig. S14**). However, the high-fat diet significantly enhanced the *P. copri-*induced host intestinal barrier permeability and chronic inflammatory reaction (*P* < 0.05; **Additional file** **1****: Fig. S14**)

## Discussion

Accumulated evidences have indicated that gut microbiota may contribute to host fat accumulation. In this study, we have identified *P. copri* from the gut microbiome of pigs fed with formula diets as a hub bacterial species increasing fat accumulation of pigs. *P. copri* is a complex comprising several distinct clades [33]. It has been both positively and negatively associated with host health depending on habitual diets. For example, *P. copri* colonization in mice fed a fiber-rich diet improved glucose homeostasis via intestinal gluconeogenesis [35, 36]. *Prevotella* abundance or the *Prevotella*-to-*Bacteroides* ratio can predict body weight and fat loss success in overweight participants consuming a whole-grain or high-fiber diet [37, 38]. However, a clinical trial report showed that a higher relative abundance of Prevotellaceae and Veillonellaceae along with increased gut permeability elevated circulating succinate levels associated with obesity and impaired glucose metabolism [39]. Prevalence of *P. copri* in the feces and plasma interleukin-6 levels were increased in type 2 diabetes patients [40]. *P. copri* is associated with human insulin resistance and aggravating glucose intolerance [15]. Different habitual diets lead to distinct genetic and functional traits of human intestinal *P. copri* strains [32], and human intestinal *P. copri* isolates show distinct polysaccharide utilization profiles [41]. In the modern pig industry, to exploit the maximum pig growth potential, commercial formula diets that are processed and contain high amounts of digestible energy and protein are provided to pig herds. These diets have selected and shaped gut *P. copri* of commercial pigs. Indeed, compared to Duroc pigs and crossbred DLYs under industrial pig husbandry settings where the animals were fed a formula diet with high content of digestible energy and protein, wild boars and Tibetan pigs fed high-fiber diets had significantly lower abundances of *P. copri*. Furthermore, from the gavage experiment with germ-free mice, we observed significantly higher abundances of colonized *P. copri* isolated in mice fed a high-fat diet. The gut microbiome of Duroc pigs from the discovery cohort had a higher abundance of *P. copri* than pigs from the validated cohort. Within a cohort the same formula feed was provided to the pigs, but we observed significant variation of the gut *P. copri* abundances in both experimental cohorts. This could have been caused by maternal effects [42] or/and the diets [43] before performance measurement (from birth to 30 kg of body weight). All experimental pigs in both cohorts were from different farms with different environments and management patterns before performance measurement. The digestible energy and crude protein of formula diets for the discovery cohort was higher than those provided to validation Duroc pigs (3,023 vs. 2,960 kcal/kg, and 17% vs. 15%). Furthermore, these pigs were genetically unrelated (from different sows). Host genetics may be another reason causing this significant variation of *P. copri* abundance in the gut [44].

Serum concentrations of LPS, LBP, BCAA, AAA, and the metabolites related to arachidonic acid metabolism were significantly higher in fat pigs than in lean pigs. Previous reports in mice have also indicated the role of gut bacterial LPS in obesity [45, 46]. Several studies in humans indicated that *P. copri* largely drives the increase of the microbial potential for BCAA biosynthesis [15], and these studies have suggested a causative role for serum level of BCAAs or their breakdown products in type 2 diabetes [47], obesity [48], and insulin resistance [15]. Arachidonic acid is the substrate for the synthesis of a range of biologically active compounds, including prostaglandins and leukotrienes [18]. These compounds can act as mediators and regulators of inflammatory cytokine production and immune function [18]. As for AAAs, increased circulating concentration of AAAs has been reported to be associated with obesity and insulin resistance in humans [47, 49, 50]. The correlation between the LMP-associated bacterial taxa and serum metabolites in pigs suggested that the gut microbiota, especially *P. copri*, drives elevated levels of serum BCAA, AAA, and the metabolites of arachidonic acid, implying that the gut microbiota should induce chronic inflammatory response via these metabolites, thereby resulting in host fat accumulation.

In contrast, the bacterial species that have been reported to have anti-inflammatory effects in humans were significantly enriched in lean pigs, including *F. prausnitzii* [51] whose abundance in the gut was negatively correlated with *P. copri* in both experimental pig cohorts (**Additional file** **1****: Fig. S15**). The function term of metabolism of cofactors and vitamins was enriched in the gut microbiome of lean pigs. Interestingly, vitamins K, D3, pantothenic acid, and biotin, which have been reported to be associated with decreased common obesity [52], reduced inflammation [53], and increased energy expenditure and adiponectin expression [54], were enriched in the serum of lean pigs.

In our previous study, we found that the *Prevotella*-predominant enterotype had a higher average daily feed intake than the *Treponema* enterotype in 280 Duroc pigs that were also included in the discovery cohort of this study. *Prevotella* (mainly *P. copri*) may be the keystone bacteria species associated with host feed intake [55]. Overall, combining the results in pigs from this study and Yang et al*.* (2018) [55] and the results from *P. copri-*colonized mice, we propose a model of gut microbiome influence on host fat deposition: (1) high abundance of *Prevotella*, especially *P. copri* in the gut may be associated with excessive energy uptake; (2) increased concentrations of serum LPS, BCAA, and arachidonic acid metabolites contributed by *P. copri* activate the *TLR4* and mTOR signaling pathways and result in host chronic inflammatory response; and (3) the genes related to lipogenesis and fat accumulation are upregulated, while the genes associated with lipolysis, lipid transport, and muscle growth are downregulated. This should increase fat accumulation and lower the LMP.

## Conclusions

In conclusion, we identified and confirmed that *P. copri* from the gut microbiome of pigs fed commercial formula diets significantly increased the fat deposition of pigs. High abundance of gut *P. copri* activates host chronic inflammation responses by the metabolites through *TLR4* and *mTOR* signaling pathways, and results in fat accumulation. The results provided fundamental knowledge for reducing fat accumulation and increasing the LMP in pigs through regulating the gut microbial composition in the pig industry, and give reference insights for the influence of gut microbiome in human obesity.

## Methods

### Experimental animals and sampling

Two experimental pig cohorts were used in this study. The discovery cohort comprised 550 Duroc pigs from Shahu (280 pigs) and Jiangying (270 pigs) farms in southern China. Another 148 Duroc pigs from the Jiangying farm were used as the validation cohort. All experimental pigs were raised under similar feeding and management conditions. The commercial formula feeds provided to experimental pigs of each farm contained 60% corn, 15% soybeans, 10% wheat bran, and 8% rice polishing. The main nutrient components of the diets are listed in **Additional file** **2****: Table S8**. Diet and water were offered ad libitum. Backfat thickness and transection area of the longissimus dorsi muscle were measured in the middle of the last 3rd and 4th ribs using a B-model ultrasound instrument (Pie-Medical, Netherlands) when the body weight of experimental pigs achieved 120 ± 10 kg, around the age of 160 ± 10 days. The GPS software was used to adjust the backfat thickness and transection area of the longissimus dorsi. Lean meat percentage (LMP) was calculated by the model: adjusted PPL = [80.95 − (16.44*adj.bf) + (4.693*adj. LMA)]*0.54 [56], where PPL represents lean meat percentage, and adj.bf and adj.LMA represent adjusted backfat thickness and transection area of the longissimus dorsi, respectively. The fecal samples were collected from all experimental pigs at the age of 160 days, conserved in sterilized tubes, and immediately immersed in liquid nitrogen for transportation and then stored at – 80 °C until use. In the validation cohort, we chose 16 fecal samples with extreme phenotype values for metagenomic sequencing, including eight samples with high LMP values (57.83 ± 0.54, mean ± SD) and eight samples with low LMP values (54.57 ± 0.59). To investigate the abundance of *P. copri* isolated in this study in the gut of pigs fed diets with different fiber contents, metagenomic sequencing data of six fecal samples from natural free-living wild boars (high-fiber diets, adults, exact age unknown), 13 fecal samples from semi-grazing Tibetan pigs at the age of 210 days (supplemented with potato and highland barley, high-fiber diets), and feces samples from (Landrace × Yorkshire) × Duroc pigs at the age of 126–140 days from pigs that were raised in Denmark (industrial pig growing setting) [34] were also used in this study. All experimental pigs were healthy and had not received any antibiotics, probiotics, or prebiotics within at least 2 months before sample collection.

### DNA extraction and 16S rRNA gene sequencing

Fecal microbial DNA was extracted using the QIAamp DNA Stool Mini Kit (QIAGEN, Germany) following the manufacturer’s guidelines. DNA concentration was measured with a Nanodrop-1000 (Thermo Scientific, USA), and the quality was assessed by agarose gel electrophoresis. The barcoded fusion forward primer 515F (5′-GTGCCAGCMGCCGCGGTAA-3′) and the reverse primer 806R (5′-GGACTACHVGGGTWTCTAAT-3′) were used to amplify the V4 hypervariable region of the 16S rRNA gene in the discovery cohort. The primers 338F (5′-ACTCCTACGGGAGGCAGCA-3′) and 806R (5′-GGACTACHVGGGTWTCTAAT-3′) were used to amplify the V3–V4 hypervariable region of the 16S rRNA gene in the validation cohort. The PCR amplification conditions were as follows: initial 95 °C denaturation step for 10 min, 35 cycles of 95 °C for 25 s, 55 °C for 20 s, and 72 °C for 5 min followed by a final extension for 10 min at 72 °C. All amplicons were sequenced using the paired-end method on a MiSeq platform (Illumina, USA) following the standard protocols.

The raw 16S rRNA gene sequencing data were filtered for the primer sequences, the barcodes, and the low-quality reads according to Illumina’s quality control procedure. High-quality paired-end clean reads were assembled using FLASH (v1.2.11) [57]. The USEARCH (v7.0.1090) quality filter pipeline was used to filter the putative chimeras and to choose operational taxonomic units (OTUs) at 97% sequence identity [58]. Only those OTUs that had relative abundance > 0.05% and were present in more than 1% of the experimental pigs were included for further analysis. Taxonomies were assigned for the aligned sequences using Quantitative Insights Into Microbial Ecology (QIIME, v1.80) with a Ribosomal Database Project (RDP) classifier [59].

### Construction of enterotype-like clustering

Enterotype-like clustering was performed according to the method described previously [60]. In brief, Jensen-Shannon divergence (JSD) distances were calculated based on the relative abundances of bacterial taxa at the genus level using the Partitioning Around Medoids (PAM) method. The optimal number of clusters and the groups’ robustness were evaluated with the Calinski-Harabasz (CH) index and silhouette value. Sparse Correlations for Compositional data (SparCC) was applied to determine co-abundance (positive) and co-exclusion (negative) relationships between genera based on their relative abundances, and significant correlations between bacterial genera were identified using the partial correlation and information theory (PCIT) algorithm [61]. The absolute correlations were transformed into links between two genera in the genus network, and the networks were visualized in Cytoscape (v3.4.0). The comparison of the LMP values between enterotypes was performed by Wilcoxon’s rank sum tests in the R package (v3.5.1).

### Association analysis between OTUs and pig LMP

The residuals of phenotypic values of the LMP corrected for the effects of sex and sampling batch (three and two sampling batches for discovery and validation cohort, respectively) were used for further association analysis between the LMP values and the relative abundances of OTUs. Because the relative abundances of OTUs exhibited a non-normal distribution pattern, the association analysis was performed using a two-part model as reported previously [62]. In brief, the two-part model accounts for both binary and quantitative characteristics of gut microbial abundance. The binary model (adj_p = β_1_b + e, adj_p represents LMP values adjusted for the effects of sex and batch,β_1_ is the estimated binary effect, b is a binary feature, and e refers to the residuals) describes a binomial analysis that tests for association of detecting a microbe with the LMP. The binary feature of a microbe under investigation was coded as 0 for undetected or 1 for detected in each sample. The quantitative model (adj_p=β_2_q +e, where β_2_ is the estimated quantitative effect, and q is a quantitative feature) evaluates the association between the abundances of the detected microbes and the LMP values. A meta-analysis was performed to assess the effects of both binary and quantitative models by using an unweighted *Z* method (*Z* = ∑^*k*^_*i* = 1_ *z*_*i*_/$\sqrt{k}$~*N*(0,1); *z*_*i*_ = ∅^−1^(*P*_*i*_)). The final association *P* value was set as the minimum of *P* values of binary, quantitative, and meta-analyses. In total, 1000 permutation tests were performed to correct for false positives, and a false discovery rate (FDR) < 0.01 was set as the significance threshold.

### Co-abundance group analysis of OTUs

The OTUs having relative abundance > 0.1% were used to construct CAGs. We first calculated the correlation coefficients among OTUs using the Sparse Correlations for Compositional data (SparCC) algorithm in both test and validation cohorts [63]. Then, CAGs were defined by a heat plot using the SparCC correlation coefficient matrix and Ward’s linkage hierarchical clustering through theMade4 (v3.40) package [64]. PERMANOVA was performed to assess the accuracy of clustering with 1000 permutations at *P* < 0.01 [65]. The network plot highlighting the SparCC correlations among CAGs was constructed in Cytoscape (v3.6.0) [66]. We numerically calculated the topological features and metrics of networks, including the average number of neighbors, average eccentricity, betweenness, closeness, and centralization degree to determine the hub OTUs of the network. Spearman’s correlation analysis was performed to test the correlations between CAGs and the LMP values in both test and validation cohorts.

### Metagenomic sequencing analysis

A pair-end (PE) library with an insertion size of 350 bp was constructed for each of 16 samples according to the manufacturer’s instructions (Illumina, USA). Sequencing was performed on a Novaseq 6000 platform (Illumina, USA). High-quality reads were obtained by filtering out adaptors, low-quality reads, and host genomic DNA contamination from the raw data.

We assembled the high-quality reads into contigs using the SOAPdenovo assembler(v.2.21) [67]. The USEARCH (v.7.0.1090) program was used to exclude the redundant contigs [58]. The contigs more than 300 bp in length were used to predict open reading frames (ORFs) by applying MetaGeneMark (v2.10) [68]. A non-redundant gene set containing 2,799,188 genes was constructed by excluding the redundant genes from all predicted ORFs using Cd-hit software (v4.6.1) [69]. A gene abundance profile was generated by mapping the high-quality reads from each sample to the non-redundant gene set using the screen function in MOCAT (v2.0) [70]. To assess gene richness in the high and low LMP pigs, we calculated the total gene number in each sample using the pair-oriented counting method [16]. The α-diversity (Shannon index) was calculated using the gene abundance profiles using the vegan R package (v3.5.1). Comparisons of gene counts and the α-diversity between high and low LMP pigs were performed using the Wilcoxon rank sum test. Taxonomic assignments of the predicted genes were performed using the BLAST + Lowest Common Ancestor (BLAST + LCA) algorithm based on the sequence similarity to the reference genomes in the non-redundant (NR) database [71]. Functional annotations were performed by aligning the putative amino acid sequences that were translated from the predicted genes against CAZy and KEGG databases using BLASTP [72]. Linear discriminate analysis effect size (LEfSe) was used to identify the bacterial species and function capacities of gut microbiome having significantly different abundances between high and low LMP pigs. Correlations between the LMP-associated bacterial species and the LMP-associated function capacities of gut microbiome were evaluated in the 16 samples with metagenomic sequencing data using Permutational analysis of variance (PERMANOVA) based on 9,999 permutations using the vegan package in R (v3.5.1) [12]. The significance threshold was set at FDR < 0.05. The correlation coefficient was calculated as Spearman’s rank correlation. The heatmap was plotted using the gplots package in R (v3.5.1) [73].

The metagenomic sequencing data of another 20 fecal samples from the discovery cohort were obtained in our previous study via the same method [17] and were also used in this study. The association of bacterial species with the LMP in the integrated 36 metagenomic sequencing data was analyzed by a two-part model as described above. The comparison of the abundance of *P. copri* among pigs fed diets with different fiber contents was performed with the metagenomic sequencing data of fecal samples from six wild boars, 13 Tibetan pigs, 36 Duroc pigs, and 20 DLYs (ERR1135357-ERR1135376) [34] as described above. Clean reads of each sample were aligned to the reference genomic sequence of *P. copri* obtained in this study using BWA MEM (v0.7.17-r1188) [74], and then the number of successfully assigned reads was computed using FeatureCounts (v2.0.1) [75]. The percentage of the reads mapped to *P. copri* reference genome in total clean reads was calculated for each sample and treated as the relative abundance of *P. copri* in each sample. The comparison of gut *P. copri* abundances among wild boars, Tibetan, and Duroc pigs was performed by a Wilcoxon test and visualized using the ggpubr package in R (v3.6.2).

### Isolation and culture of the bacterial strain of *P. copri* from pig fecal samples

The fecal samples from 22 experimental Duroc pigs with both extreme phenotypic values of fat accumulation (low LMP) and high abundance of *P. copri* were collected and used for the *P. copri* isolation experiment. One-gram fecal samples were suspended in phosphate buffered saline (PBS) buffer and serially diluted to 10^−8^. Eighty-microliter diluted samples were plated anaerobically on Bacteroides mineral salt agar to isolate *P. copri* [76]. The plates were incubated at 37 °C for 2–7 days in an anaerobic workstation (ELECTROTEK AW500SG, UK) filled with 80% N_2_–10% CO_2_–10% H_2_ gase s[77]. A single colony from plates was selected according to the main characteristics of the strain that we were looking for based on the previous description [78], i.e., white, circular, convex, and gram-negative rods, and purified by streaking the single bacterial colony on modified PYG agar supplemented with 5% (v/v) sterile defibrinated sheep blood with a sterile probe [78]. The plates were maintained under the culture conditions mentioned above for two days. The 16S rRNA gene of the single strain was amplified using two universal primers 27F (5'-AGAGTTTGATCCTGGCTCAG-3') and 1492R (5'-GGTTACCTTGTTACGACTT-3') and sequenced by the Sanger method. The 16S rRNA gene sequences were then aligned to the NCBI nucleotide sequence database to determine *P. copri* strains. In addition, we blasted the 16S rRNA gene sequences of the isolated strains with the V3–V4 sequence of the OTU1905 (*P. copri*) that was most significantly associated with the LMP in this study. The isolated strain with > 99% sequence identity was used for gavage in germ-free mice. The *P. copri* strain isolated above was cultivated in modified PYG medium for 36 h under anaerobic conditions, harvested in log phase, centrifuged at 1000 rpm for 10 min, and then washed twice with PBS. The precipitate was re-suspended with 5% sterile non-fat milk prepared by PBS and stored at −80°C until use.

### Whole-genome sequencing of *P. copri*

The isolated *P. copri* strain was recovered and grown on PYG liquid medium at 37°C with 80%-N_2_-10%CO_2_-10%H_2_ for 72h. Ten milliliters of cultured PYG fluid was centrifuged at 5000 rpm for 10 min. *P. copri* cells were washed twice using sterilized PBS solution and collected for DNA extraction. Genomic DNA of *P. copri* was extracted using QIAamp DNA Mini Kit according to the manufacturer’s instructions. The quantity and quality of extracted DNA were evaluated by agarose gel electrophoresis, NanoDrop-2000 (ThermoFisher, USA) and Qubit (ThermoFisher, USA).

A Nanopore sequencing library was prepared according to Oxford Nanopore’s “1D gDNA selection for long reads” protocol (Oxford Nanopore Technologies, UK). In brief, 2μg of genomic DNA of *P. copri* was sheared using a g-Tube (Covaris, USA) with 150 μl of nuclease-free water at 5000 rpm for 2 min. Long DNA fragments were enriched using the Blue Pippin selection system (Sage Science, USA). Subsequent purification of the DNA fragments was performed using AMPure beads. Nanopore 1D adapters were ligated to the end-repaired and adenylated DNA fragments using NEB Blunt/TA Master Mix (NEB, UK). The libraries were sequenced on a GridION X5 (Oxford Nanopore Technologies, UK). To improve the sequence quality, a library for second generation sequencing was prepared according to the standard protocol and sequenced on an Illumina Hiseq-2500 platform using a paired-end strategy.

Base calling of Nanopore raw data was performed with cloud-based Metrichor workflow [79]. Nanopore reads were processed using Poretools to convert fast5 files to fasta format [80]. High-quality reads were selected for further genome assembly. Canu (v1.7.11) was used for genome assembly of the Nanopore sequencing data [81]. Illumina paired-end reads were aligned and used for correcting base errors, fixing mis-assemblies, and filling gaps by Pilon (v1.22) [82]. After removing redundant sequences, the automated assembly of the *P. copri* genome was performed by Circlator (v1.5.5) [83]. To estimate the sequence contiguity and coverage, Nanopore reads after quality control were mapped to the assembled genome using Minimap2 (v2.11-r797) and Samtools (v1.9) [84, 85]. In addition, plasmid sequences were identified by blasting the genome against the plasmid database [86].

Protein-coding genes of *P. copri* genome were predicted using Prodigal (v2.6.3) [87]. The predicted protein-coding genes were further annotated with InterProScan using Blast2GOagainstPfam (release 31.0), TIGRFAMs (release 15.0) and SMART (v8.0) databases [88, 89]. Functional annotation of protein-coding genes was also performed by Blast2GO with the KEGG database. To compare the abundances of those interesting genes identified on the *P. copri* genome and participating in arachidonic acid metabolism, BCAA biosynthesis, AAA biosynthesis and metabolism, insulin resistance, and other glycan degradation between high and low LMP pigs, the sequences from the metagenomic sequencing data were mapped to the obtained *P. copri* genome. The relative abundances of these genes were determined and compared using Wilcoxon tests. FDR < 0.05 was set as the significance threshold.

### Construction of phylogenetic tree and analysis polysaccharide utilization loci of *P. copri* isolates

To construct the phylogenetic tree of *P. copri* isolates from humans and pigs, we downloaded 111 *P. copri* genomes from westernized and non-westernized human gut microbiome [33]. Gff file of each genome was generated using prokka (v1.11) [90] and used to produce the alignment of core genes by Roary (v3.11) [91]. The phylogenetic tree was constructed based on the alignments of core genes using neighbor-joining approach in Megan 7 and visualized by iTOL [92]. Polysaccharide utilization loci of *P. copri* isolates were predicted by using deCAN-PUL with identity > 75% and *E* value < 1e−50 [93].

### Mouse intervention study

Twenty-one germ-free mice having similar body weight and size (Kunming; 12 males and nine females, each 6 weeks of age) used in this study were housed in cages under sterile conditions. Male and female mice were kept separately. Feed and water were available *ad libitum*. After 2 weeks of acclimatization to the new environment and the standard chow diet, mice were randomly divided into three groups (four males and three females per group). One group received a chow diet with *P. copri* administered by gavage. A high-fat diet group (60% fat, Research Diet, D12492) was administered with *P. copri* by gavage, and a chow diet group without gavage was used as a control. For the two colonization groups, mice were given 100 μl of *P.copri* suspension (1 × 10^7^ CFUs/μl) three times a week for 4 weeks. To further investigate the effect of diet on *P. copri* colonization and host fat accumulation, we used another 18 germ-free mice with similar body weights and sizes (C57BL6; nine males and nine females) to perform gavage experiments using *P. copri*. These germ-free mice were managed and administered the bacteria using the same gavage methods and procedures described above. The 18 mice were randomly divided into three groups (three females and three males for each group) comprising a standard chow diet group, a high-fat diet (60% fat, Research Diet, D12492) group, and a high-fiber diet (35% fiber) group. The feeding experiment lasted 4 weeks, and mice were administered *P. copri* by gavage three times per week as described above.

Fecal samples were collected at the end of the gavage experiment, dipped into liquid nitrogen immediately, and stored at − 80°C until use. All mice in each group had lean mass measured and body fat percentage calculated by a whole-body composition analyzer (Niumag, China) following the manufacturer’s instructions. After the body weight measurements, all mice were sacrificed by cervical dislocation. Epididymal fat was isolated and weighted for all mice. The epididymal fat percentage (EMP) was calculated. Tissue samples of the colon, epididymal white adipose, and muscle were sampled from each experimental mouse for further RNA-seq analysis. Venous blood was taken from the inner canthus of each mouse for serum metabolomic analysis. The concentrations of lipopolysaccharide, intestinal barrier permeability plasma biomarkers, and pro-inflammatory cytokines were also determined in serum samples of phenotyped mice by ELISA using the method described above.

### Quantifying the abundances of *P. copri* in treated mice

Mouse fecal bacterial DNA was extracted using the QIAamp fast DNA stool mini kit (Qiagen, Germany) as described above. The quantitative PCR was performed using a 7500-Fast Real-Time PCR System (ABI, USA) and SYBR® Premix Ex Taq™ II (TaKaRa, Japan). The two-step real-time PCR conditions were described as follows: an initial denaturation for 10 s at 95 °C, 40 cycles of denaturation at 95 °C for 5 s, and annealing at 60 °C for 25 s. The RQ value of *P. copri* was determined by normalization to the 16S rRNA gene using the2^−ΔΔCt^ method [15]. Primer sequences are listed in **Additional file** **2****:** Table S9 [94].

### Determination of metabolome profiling of serum samples

Metabolome profiles of serum samples were determined for 38 pigs randomly selected from the validation cohort, and for seven mice from each of control, *P. copri* gavage, and *P. copri* gavage + HFD feeding groups (a total of 21 samples). Blood samples were collected from the anterior vein. After being placed into serum separator tubes, all samples were centrifuged at 2500 × g for 15 min at room temperature to isolate the serum. Serum samples were immediately stored at – 80 °C until use. A 100-μL aliquot of serum sample was used for the extraction of metabolites using 3 ml of pre-cooled methanol (chromatographically pure) (Merck Corp., Germany). After vortexing for 1 min and incubation at – 20 °C in a refrigerator for 3 h, the mixture was centrifuged at 15,000 rpm for 15 min at 4 °C to precipitate the protein. Then, 200 μl of the supernatant was processed in a Speedvac overnight. The concentrated product was resuspended by the addition of 150 μl of water/methanol (85:15, v/v) and then placed into a sampling vial pending ultraperformance liquid chromatography-quadrupole time-of-flight mass spectrometry (UPLC-QTOFMS) (Waters Corp., USA). The quality control was performed via a pooled QC sample by mixing equal volumes (15 μl) of each serum sample.

Chromatographic separations were performed on a UHPLC BEH C18 column (2.1 mm × 100 mm, 1.7 μm) (Waters Corp., USA) maintained at 40°C. The injection volume was 0.4 μl for each sample, and the samples for blank-QC-tests were run alternately. The column was eluted with a linear gradient of 1–20% B at 0–3 min, 20–50% B at 3–5 min, 50–70% B at 5–10 min, 70–85% B at 10–15 min, and 85–100% B at 15–17 min followed by a re-equilibration step of 5 min. For electrospray positive ion mode (ES+) analysis, the mobile phase was water with 0.1% formic acid (A) and acetonitrile with 0.1% formic acid (B). For negative ion mode (ES−) analysis, eluents A with water and B with acetonitrile were used. The flow rate was set at 0.3 mL/min. All the samples were kept at 8 °C during the analysis.

The mass spectrometric data in both positive and negative modes were collected using an electrospray ionization source. The source parameters were set as follows: capillary voltage: 3 kV; drying gas flow: 11 L/min, and gas temperature: 350 °C. Centroid data were collected from 50 to 1200 m/z with a scan time of 0.3 s and an interscan delay of 0.02 s over a 20-min analysis time. MassLynx software (Waters, USA) was used for system controlling and data acquisition. Data normalization was performed by QC samples using MetNormalizer in R (v 3.5.1) that generated a data matrix containing retention time, m/z value, and normalized abundance [95]. To obtain metabolite names and molecular formulas, we aligned the molecular mass data (m/z) of ions to the metabolites in the HMDB database with a mass error of 10 ppm or less [96].

Associations between serum metabolites and porcine LMP phenotypic values were tested by Spearman rank correlation in the 38 experimental pigs. The analysis was performed using both 16S rRNA gene sequencing and metabolome analyses at FDR < 0.05. The correlations between the LMP-associated serum metabolites and the LMP-associated OTUs were assessed by Spearman correlation coefficients (FDR < 0.05). To further evaluate the correlation between the LMP-associated bacterial species and the LMP-associated serum metabolites in the 16 tested samples from the metagenomic sequencing, the metabolites differing in normalized abundance between high (*n* = 8) and low LMP pigs (*n* = 8) were identified by LEfSe. The online MetaboAnalyst program was used to assign the differential metabolites to KEGG pathways [97]. PERMANOVA and Spearman’s correlation analysis were performed to assess the correlations between the LMP-associated bacterial species and the LMP-associated serum metabolites as described above. The serum metabolites with different abundances between controls and *P. copri*-gavaged mice were identified by LEfSe.

### Quantifying serum concentrations of lipopolysaccharide, intestinal barrier permeability biomarkers, and pro-inflammatory cytokines

We quantified the concentrations of serum lipopolysaccharide (LPS), lipopolysaccharide binding protein (LBP), fatty acid-binding protein 2 (FABP2), zonulin, diamine oxidase (DAO), IL-1β, IL-6, IFN-γ, and TNF-α using the enzyme linked immunosorbent assay (ELISA) method with commercial ELISA kits (CUSABIO, China) following the manufacturer’s instructions. Briefly, except for the blank control wells, 50 μl of standard samples or appropriately diluted serum samples were added into the 96-well microtiter plates coated with the primary antibodies, and then 100 μl of HRP-conjugated secondary antibodies was added to the microtiter plates and incubated for 60 min at 37 °C. Microtiter plates were washed four times with washing buffer, and 50 μl of substrates A and B were added to each well of microtiter plates, mixed gently, and incubated for 15 min at 37 °C under light shading conditions. Finally, 50 μl of enzymatic reaction termination solution was added to each well to stop the reaction. The O.D. value for each sample at 450 nm was measured and recorded using a microtiter plate reader (Tecan Infinite 200 pro, Switzerland). A standard curve was plotted according to the O.D. values and the concentrations of standard samples. The serum concentrations of LPS, LBP, intestinal barrier permeability plasma biomarkers, and pro-inflammatory cytokines in each test sample were determined using the standard curve. Each standard and tested serum sample was measured in triplicate. The Wilcoxon rank sum test was used to compare the serum concentrations of LPS, LBP, FABP2, zonulin, DAO, and pro-inflammatory cytokines between high and low LMP pigs at an FDR < 0.05. The multiple group comparisons of these data among experimental mice were performed by the Kruskal-Wallis test^34^. All these analyses were carried out using the R software (v3.5.1).

### RNA extraction, sequencing, and data analysis

The mice used for confirming the causality of *P. copri* were further used for RNA sequencing analysis. Six mice from the group administrated with *P. copri* and fed standard chow diet, and the other six mice from the control group were randomly chosen. Total RNA was extracted from colon, epididymal white adipose, and muscle tissues using Trizol (ThermoFisher, USA) according to the manufacturer’s manuals. The RNA concentration and integrity were assessed using a Nanodrop-1000 spectrophotometer (ThermoFisher, USA) and a bioanalyzer-2100 (Agilent, USA). The cDNA libraries were prepared using the Illumina Truseq Stranded mRNA preparation kit (Illumina, USA) according to the manufacturer’s guidelines. The libraries were sequenced on an Illumina HiSeq 2500 platform (Illumina, USA). Raw data were trimmed for adapter sequences, and low-quality reads were filtered out to generate clean data. After that, the HISAT, StringTie and Ballgown pipelines were used to explore differentially expressed genes (DEGs) between controls and colonized mice as described previously [98]. Briefly, Hisat2 (v2.1.0) was employed to build a reference genome index, and then high-quality read sequences were aligned to the mouse reference genome assembly (GRCm38) to generate SAM files. Samtools (v1.8.0) was used for SAM file transformation and read sorting to generate sorted bam files [84]. Transcript assembly and quantification were performed using StringTie (v1.3.4). The outputs of StringTie, including gene annotation and gene abundance files, were processed by Ballgown (v3.5) to identify DEGs based on FPKM values with FDR < 0.05.

### Statistical analysis

Shapiro-Wilk’s and Levene’s tests were performed to evaluate the distribution and equality of variances of the LMP values in the tested pig populations. The significance levels of differential LMP values between two groups of pigs selected for metagenomic sequencing (8 vs. 8), and between two groups of pigs used for determining serum metabolome profiles (17 vs. 21) were determined by *t*-tests. All analyses were performed using R (v3.5.1). The bacterial species, function capacities of gut microbiome, and serum metabolite features showing differential abundance between high and low LMP pigs were identified using LEfse using the online version of Galaxy at a significance threshold criteria of LDA score > 2.5 and alpha value < 0.01 [99]. The associations between the relative abundances of bacterial species and serum metabolite features were analyzed with the PERMANOVA method. The correlations between the LMP-associated bacterial species and the LMP-associated functional capacities of gut microbiome, and between the LMP-associated bacterial species and the LMP-associated serum metabolites, were evaluated using Spearman’s correlation at FDR < 0.05.

For the colonization experiments using mice, we first tested the distributions of the phenotypic data in each group by the Shapiro-Wilk and Levene’s tests. The multiple group comparisons of the phenotypic values of body fat percentage and epididymal fat percentage among controls, *P. copri-*gavaged mice, and HFD + *P. copri-*gavaged mice or among *P. copri*-gavaged, *P. copri*-gavaged + HFD and *P. copri*-gavaged + high-fiber diet groups were performed by Tukey’s HSD tests at FDR < 0.05. Differential serum metabolites between groups were identified by LEfSe at LDA score >3.5 and alpha value < 0.01.

## Supplementary Information

**Additional file 1.**

**Additional file 2.**

**Additional file 3.**

## Data Availability

The 16S rRNA gene sequencing data of the discovery cohort were submitted to the SRA database in NCBI with the accession number PRJNA356465. The 16S rRNA gene sequencing data of the validation cohort, metagenomic sequencing data, RNA sequencing data of experimental mice, and the whole-genome sequence of the *P. copri* isolate were submitted to China National GeneBank Database (CNGBdb) with accession code CNP0000828, CNP0000824, CNP0001725 and CNP0001731, respectively. The genome sequences of 111 *P. copri* isolates from westernized and non-westernized human populations were downloaded from http://segatalab.cibio.unitn.it/data/Pcopri_Tett_et_al.html. The codes and software for multi-omics analysis are described in Additional file 3: code and software availability.

## Abbreviations

AAAs: Aromatic amino acids
BCAAs: Branch chained amino acids
*B. longum*: Bifidobacterium longum
BP: Biological process
CAGs: Co-abundance groups
CAZymes: Carbohydrate-active enzymes
DGEs: Differentially expressed genes
ELISA: Enzyme-linked-immunosorbent serologic assay
EMP: Epididymis fat percentage
FABP2: Fatty acid-binding protein 2
FDR: False discovery rate
FPKM: Reads per kilobase of exon model per million mapped reads
IFN-γ: Interferon-γ
IL-1β: Interleukin-1β
IL-6: Interleukin-6
LEfse: Linear discriminant analysis effect size
LPS: Lipopolysaccharide
LBP: Lipopolysaccharide-binding protein
DAO: Diamine oxidase
KEGG: Kyoto encyclopedia of genes and genomes
OTU: Operational taxonomic unit
PULs: Polysaccharide utilization loci
SparCC: Sparse Correlations for Compositional data
TNF-α: Tumor necrosis factor alpha
