# Supplementary material for: Prevotella copri increases fat accumulation in pigs fed with formula diets
Source: Microbiome. 2021 Aug 21;9:175. doi: 10.1186/s40168-021-01110-0 (PMC8380364; doi:10.1186/s40168-021-01110-0)
Supplement: Supplementary file 2 — Additional file 1. [file 40168_2021_1110_MOESM1_ESM.docx]

***Prevotella copri* increases fat accumulation in pigs fed by formula diets**

Congying Chen ^a 1†^, Shaoming Fang ^a 1^, Hong Wei^2^, Maozhang He^1^, Hao Fu^1^, Xinwei Xiong^1^, Yunyan Zhou^1^, Jingyuan Wu^1^, Jun Gao^1^, Hui Yang^1^, Lusheng Huang^1†^

1. *State Key Laboratory of Pig Genetic Improvement and Production Technology, Jiangxi Agricultural University, Nanchang, 330045, China;*

2. *State Key Laboratory of Agricultural Microbiology, College of Animal Sciences and Technology, Huazhong Agricultural University, Wuhan, 430070, China*

**Running title:** *P. copri* increases fat accumulation of pigs

a. These authors contribute equally to this work

† **Correspondence authors**

Lusheng Huang, Congying Chen

State key Laboratory for Pig Genetic Improvement and Production Technology

Jiangxi Agricultural University, Nanchang, 330045, P. R. China

Phone: 0086-791-83813080

Fax: 0086-791-83900189

E-mail: Lushenghuang@hotmail.com (Lusheng Huang);

chcy75@hotmail.com (Congying Chen)

**
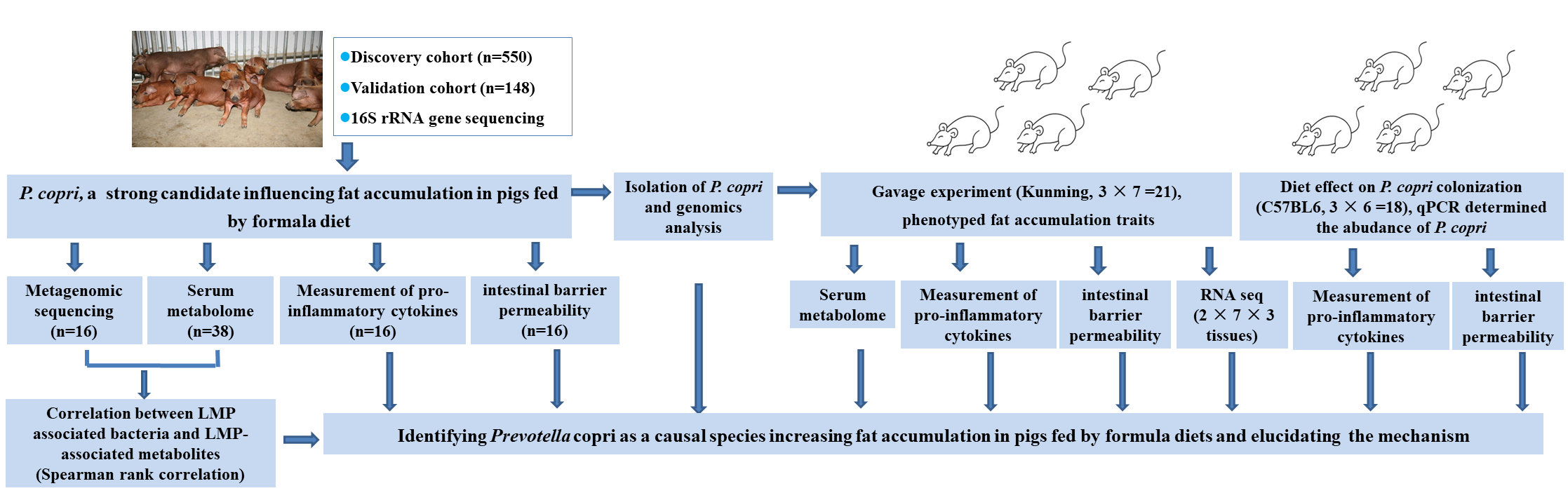
**

**Supplementary Figure S1. The flowchart of this study.**


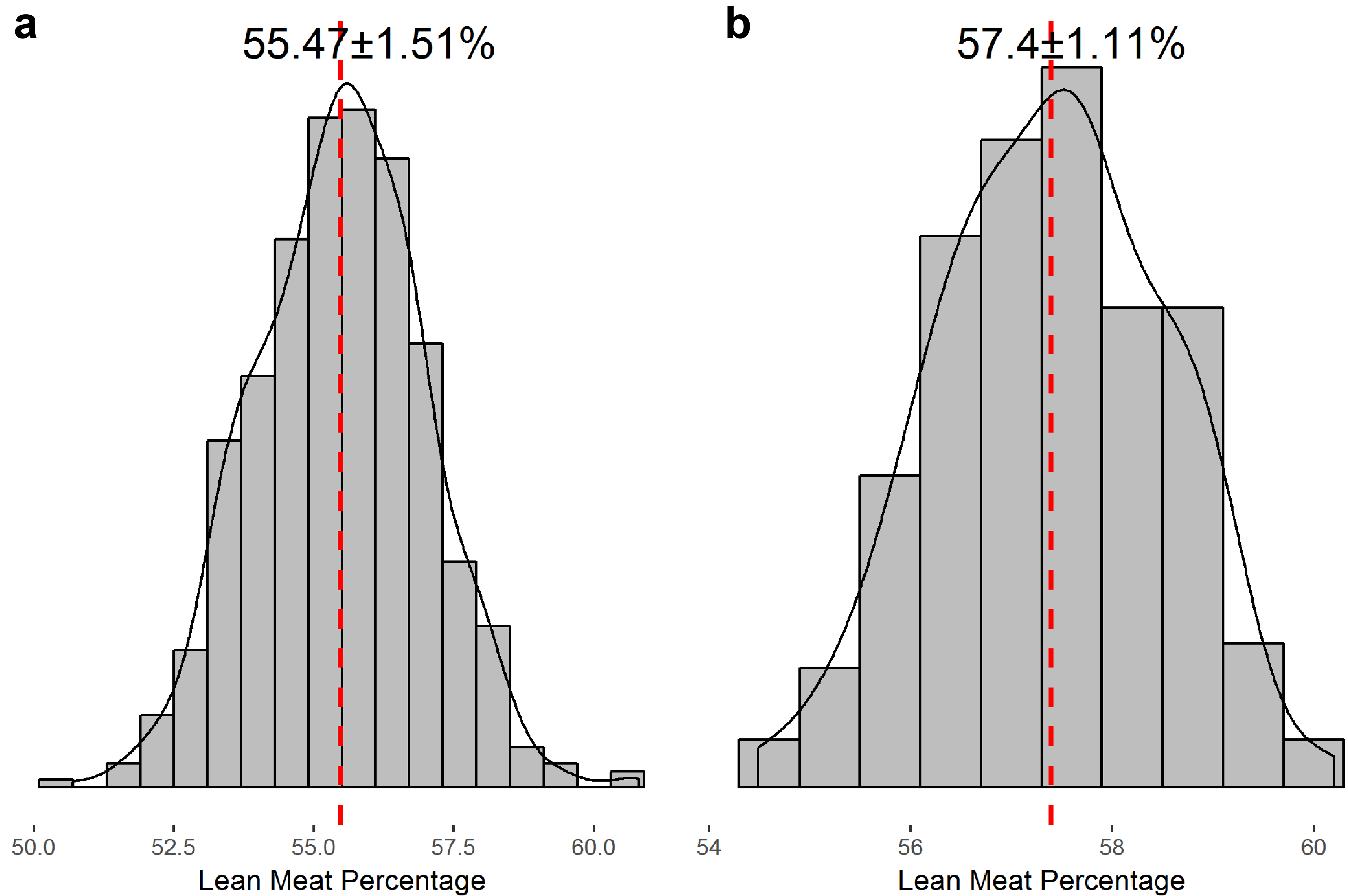


**Supplementary Figure S2. The distribution of phenotypic values of lean meat percentage in the discovery cohort** **(a) and the validation cohort (b).** The phenotypic values of lean meat percentage in both cohorts obey a normal distribution. The mean ± SE and CV of phenotypic values are directly shown in the figures.


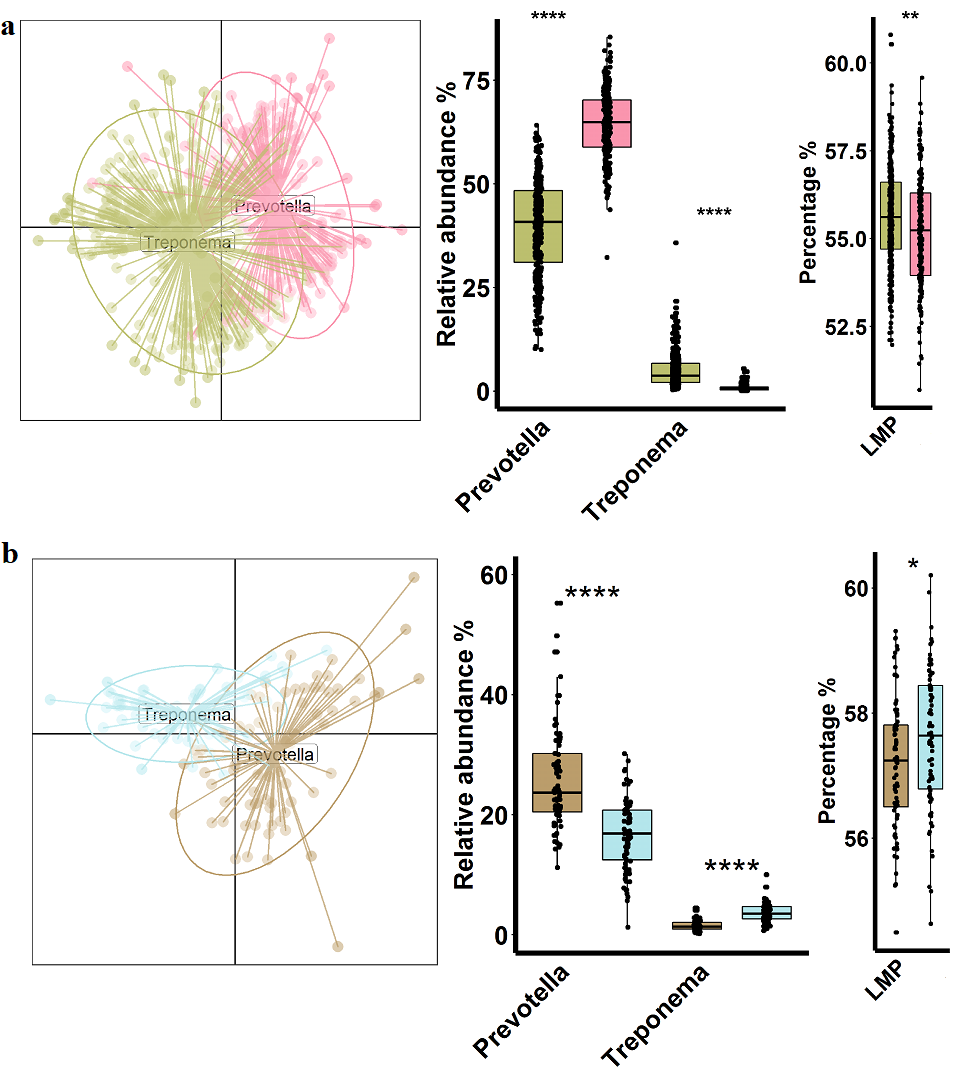


**Supplementary Figure S3. Enterotypes and its association with lean meat percentage in the discovery and validation cohort.** (a) PCoA plot showing the two enterotypes in the discovery cohort. The samples were clustered into two enterotype-like groups, which were dominated by either *Prevotella* (pink) or *Treponema* (yellow). (b) The relative abundance of *Prevotella* and *Treponema* in two enterotypes. (c) The pigs with *Prevotella* enterotype had significantly higher lean meat percentage compared with those with *Treponema* enterotype. (d-f) The enterotypes in the validation cohort. The legends were as same as that for (a-c). *, *P* < 0.05; ****, *P* < 0.001.


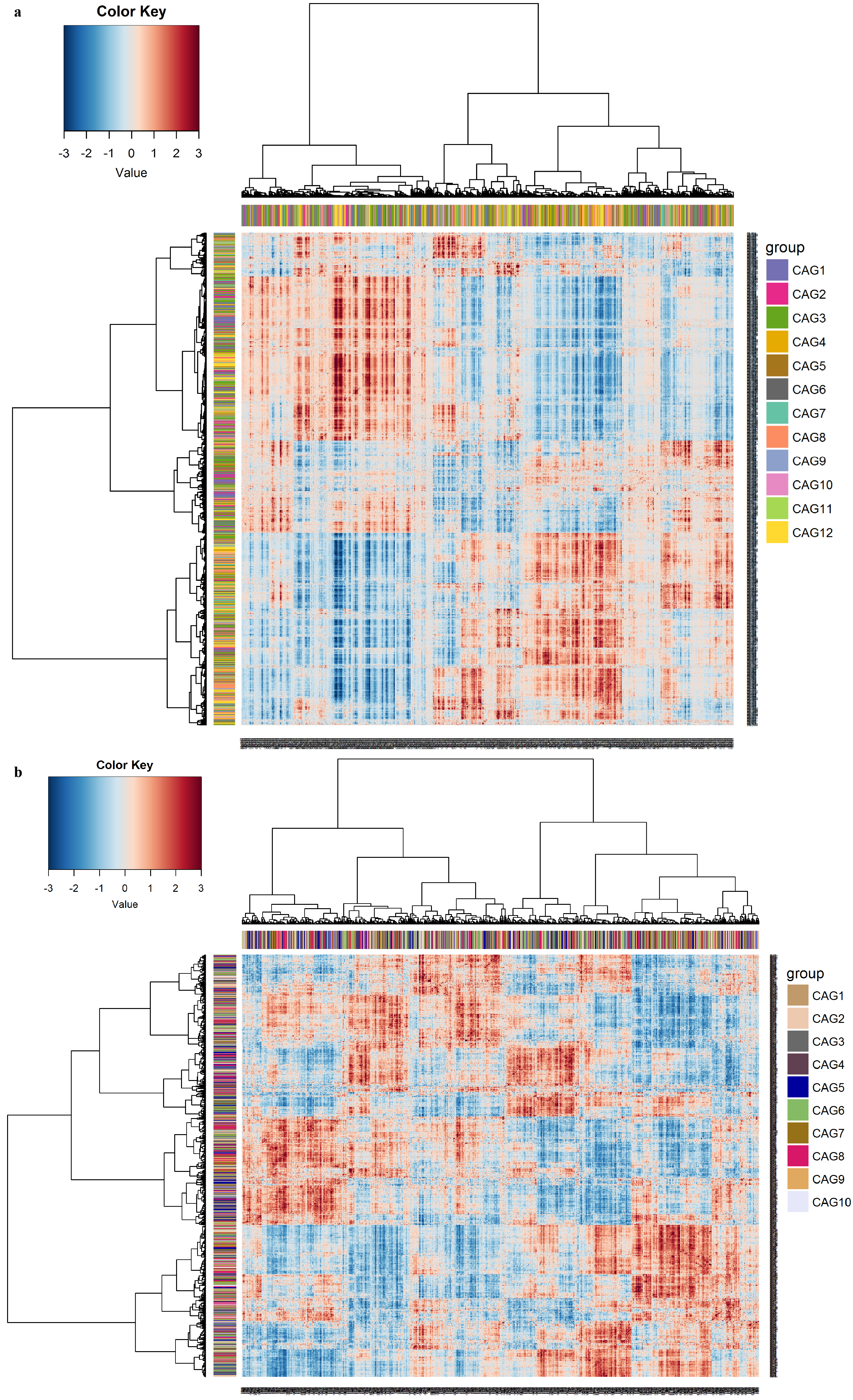


**Supplementary Figure S4. The clustering of the OTUs passed quality control and used for constructing co-abundance groups (CAGs).** (A) The 1,159 OTUs are clustered into 12 CAGs in the test cohort. (B) The 883 OTUs are clustered into 10 CAGs in the validation cohort. PERMANOVA was performed to assess the accuracy of clustering with 1000 permutations at P < 0.01.


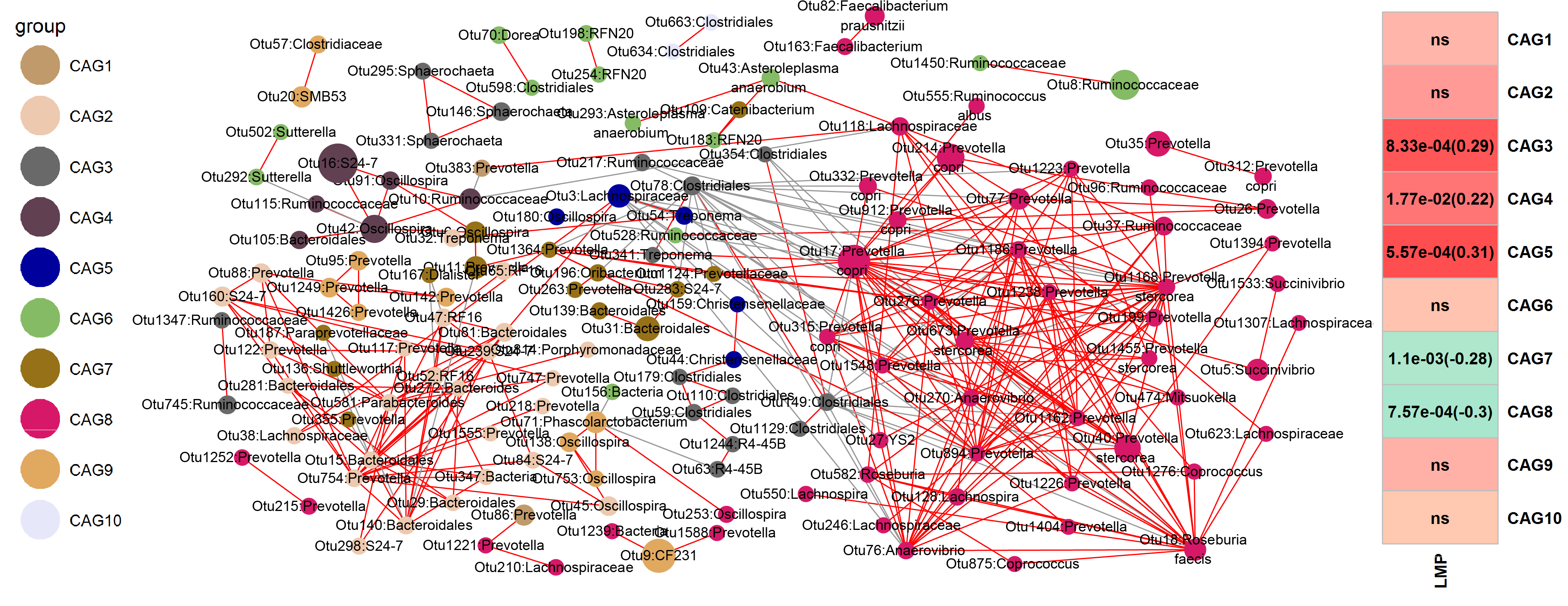


**Supplementary Figure S5. Co-abundance group analysis of lean meat percentage-associated OTUs in the validated cohort.** Network diagram of the 883 OTUs. Node size shows the average abundance of each OTU. The red and grey lines between nodes indicate the positive and negative correlations between the nodes, respectively. Only lines corresponding to the correlations with a magnitude greater than 0.5 are drawn. The OTUs are grouped into 10 co-abundance groups (CAGs) by permutational multivariate analysis of variance (PERMANOVA) when *P* < 0.01. The bars from light blue to red on the right show the P values and coefficients (in the brackets) of the correlations between CAGs and lean meat percentage (LMP).

**
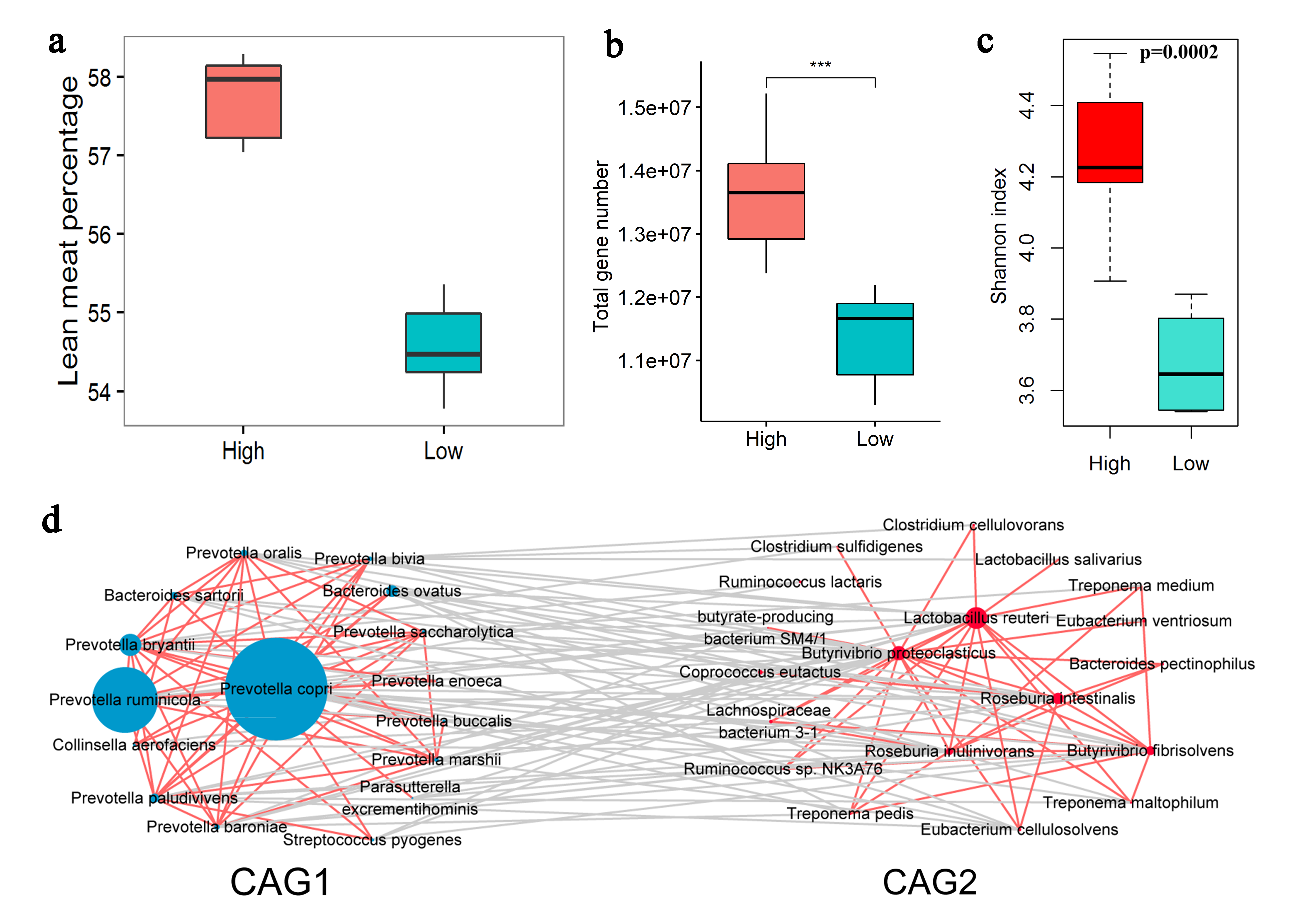
**

**Supplementary Figure S6. The phenotypic values, α-diversity and co-abundance network of the lean meat percentage (LMP)-associated bacterial species in the samples used for metagenomic sequencing.** High means the high LMP pigs (lean pigs, *n* = 8), and low indicates the low LMP pigs (fat pigs, *n* = 8) (**a**) The distinct phenotypic values of lean meat percentage between two groups of pigs whose fecal samples were used for metagenomic sequencing. **b**, Comparison of gene counts in the gut microbiome between lean (*n* = 8) and fat individuals (*n* = 8) with metagenomic sequencing data. Lean pigs were identified the higher gene number in the gut microbiome compared to obese pigs. **c**, Comparison of Shannon index of gut microbiome between fat (*n* = 8) and lean pigs (*n* = 8) at the species level. Lean pigs had the higher α-diversity of gut microbiota. **d**, Species-level network diagram of the 40 species responding to porcine LMP with metagenomic sequencing data. Node size shows the average abundance of each species. Lines between nodes indicate the correlations between the nodes, with red and grey colors indicating positive and negative correlation, respectively.


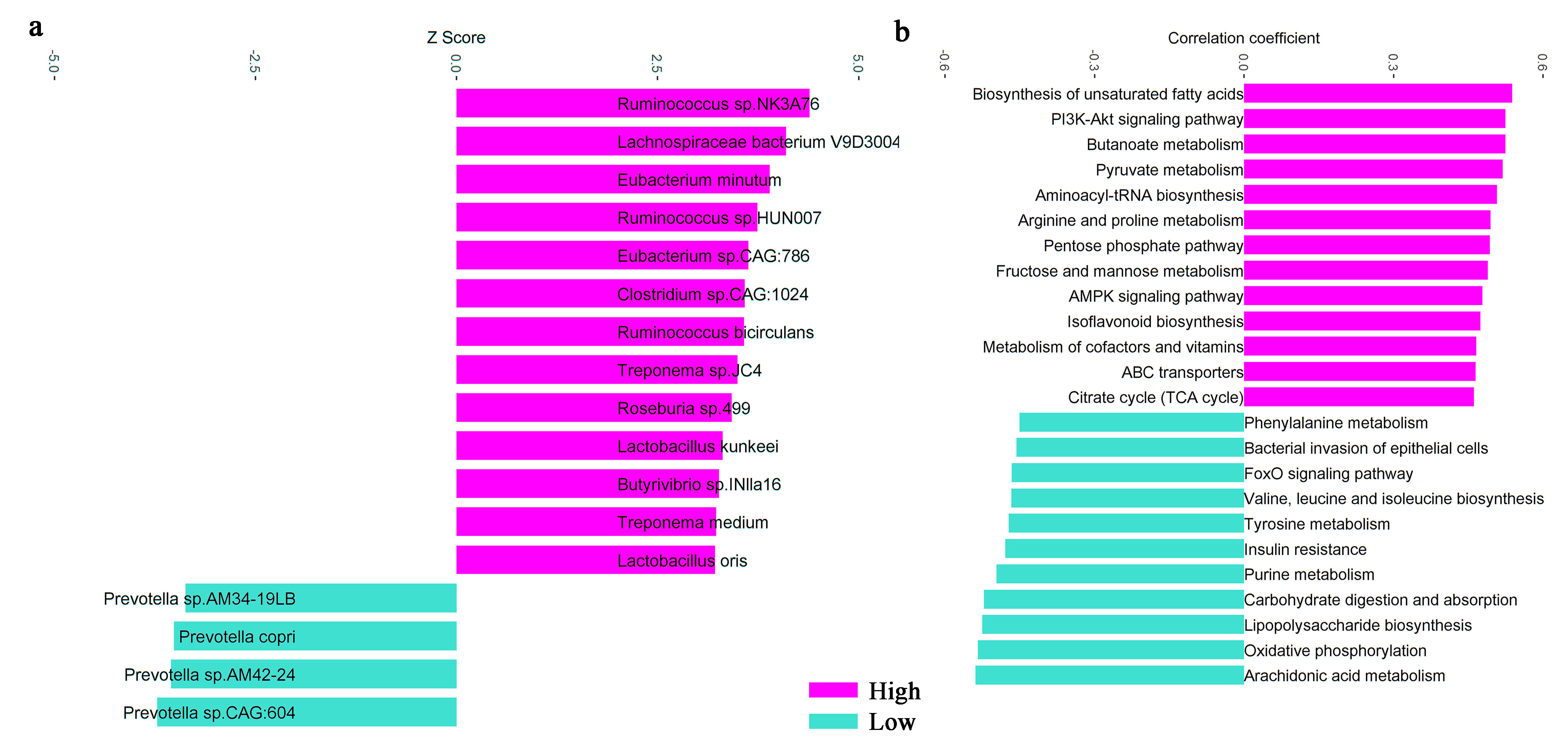


**Supplementary Figure S7. The bacterial species and KEGG pathways associated with the lean meat percentage (LMP) using** **the integrated 36 metagenomic sequencing data.** (a) The bacterial species associated with the LMP in 36 metagenomic sequencing data by two-part model analysis. The *x*-axis shows the Z-score obtained in the two-part model analysis. b, The KEGG pathways associated with the LMP in 36 metagenomic sequencing data by spearman rank correlation analysis. The *x*-axis shows the correlation ecoefficiency obtained in the correlation analysis. The red bars show the higher abundances of bacterial species and KEGG pathways in high LMP pigs, and the blue bars indicate the higher abundances of bacterial species and KEGG pathways in low LMP pigs (obese pigs).


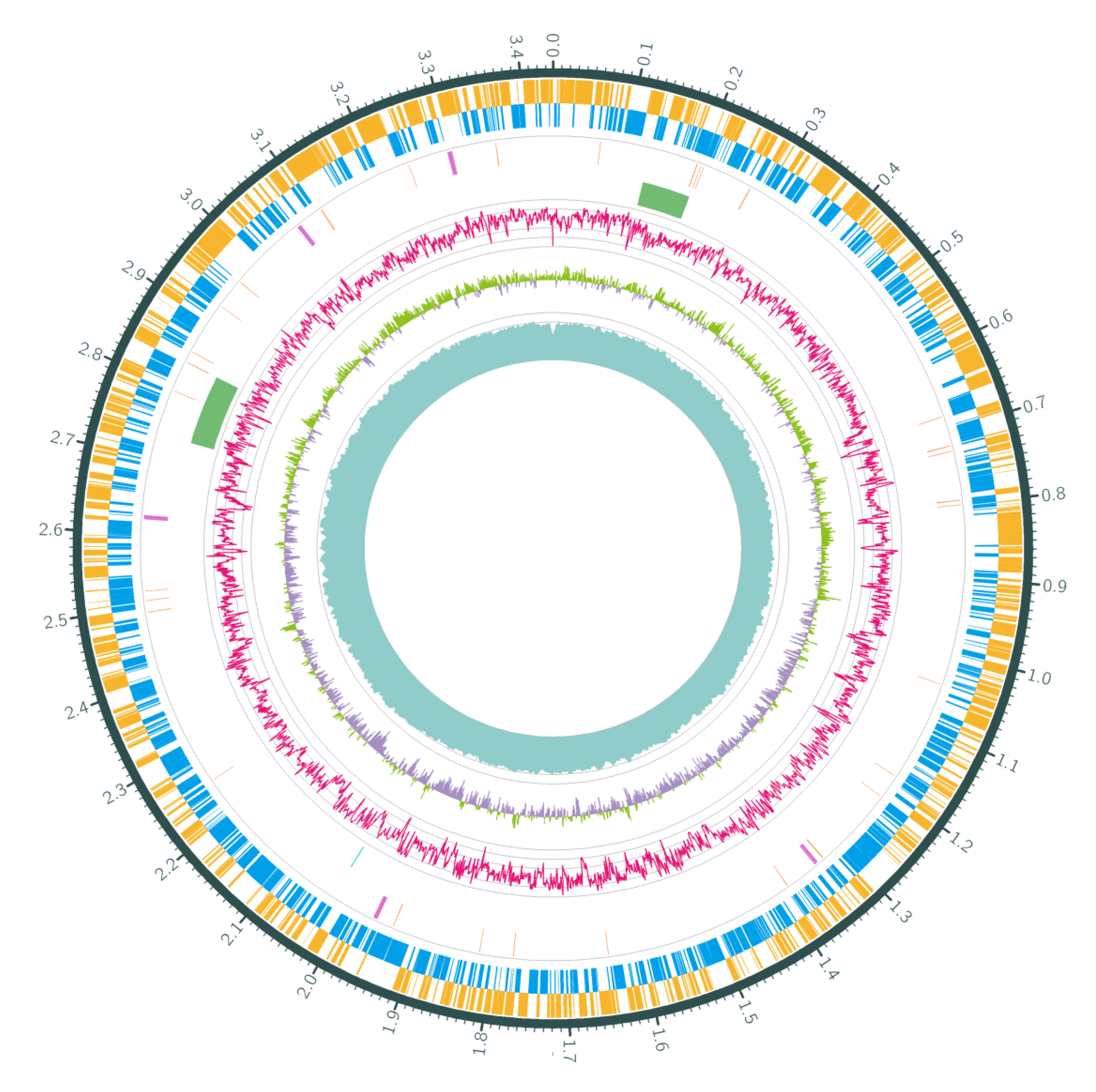


**Supplementary Figure S8.** **The map of the *P. copri* genome obtained by Nanopore sequencing.** The cycles from the outside represent: encoding gene (Sense strand), encoding gene (Antisense strand), tRNA (orange) and rRNA (purple), CRISPR (blue) and gene island (green), GC ratio, GC -skew, and sequencing depth.


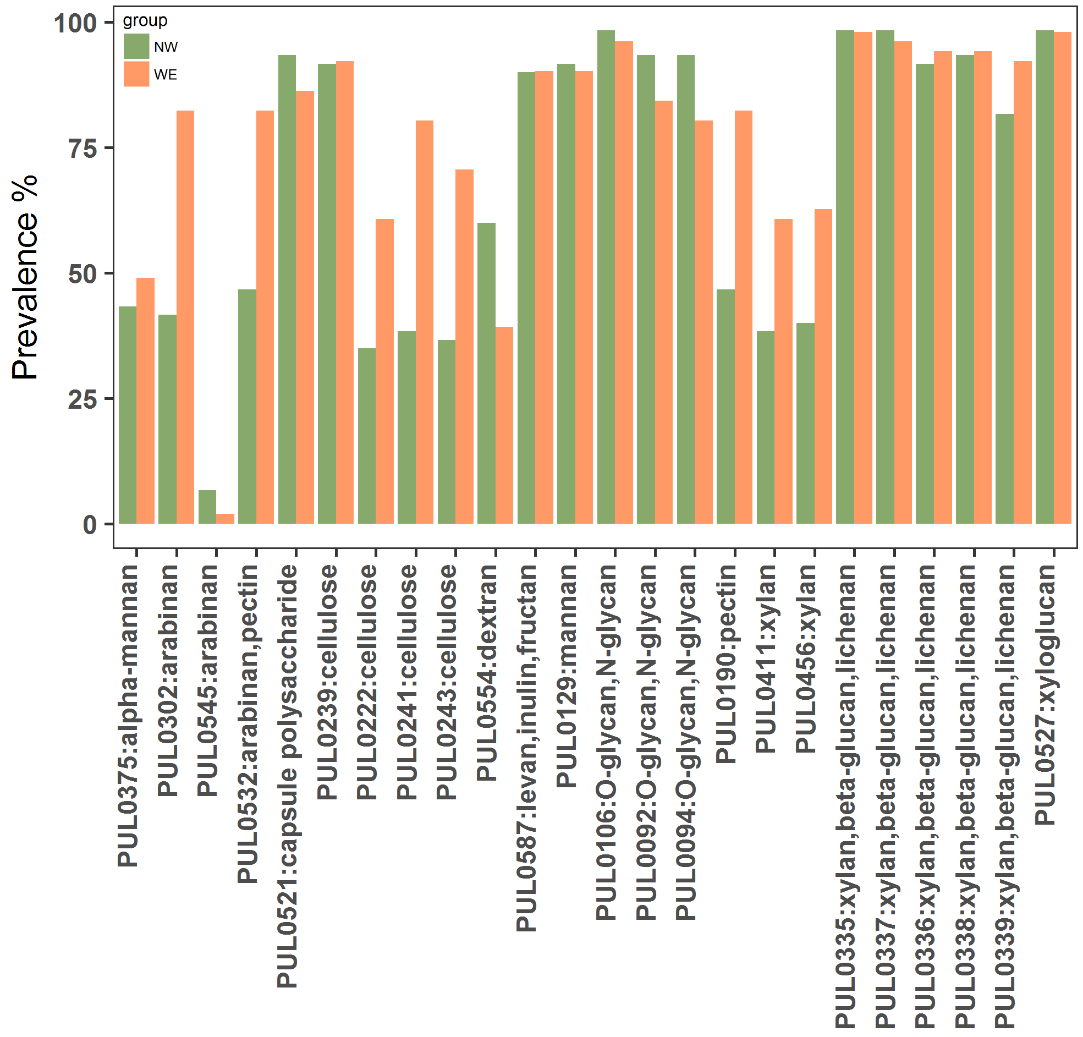


**Supplementary Figure S9. Polysaccharide utilization loci (PULs) identified in the genome of *P. copri* isolated in this study.** A total of 24 PULs were identified in this *P. copri* isolate. The PULs related to catabolism of arabinan, cellulose, pectin and xylan had higher prevalence in westernized peoples.


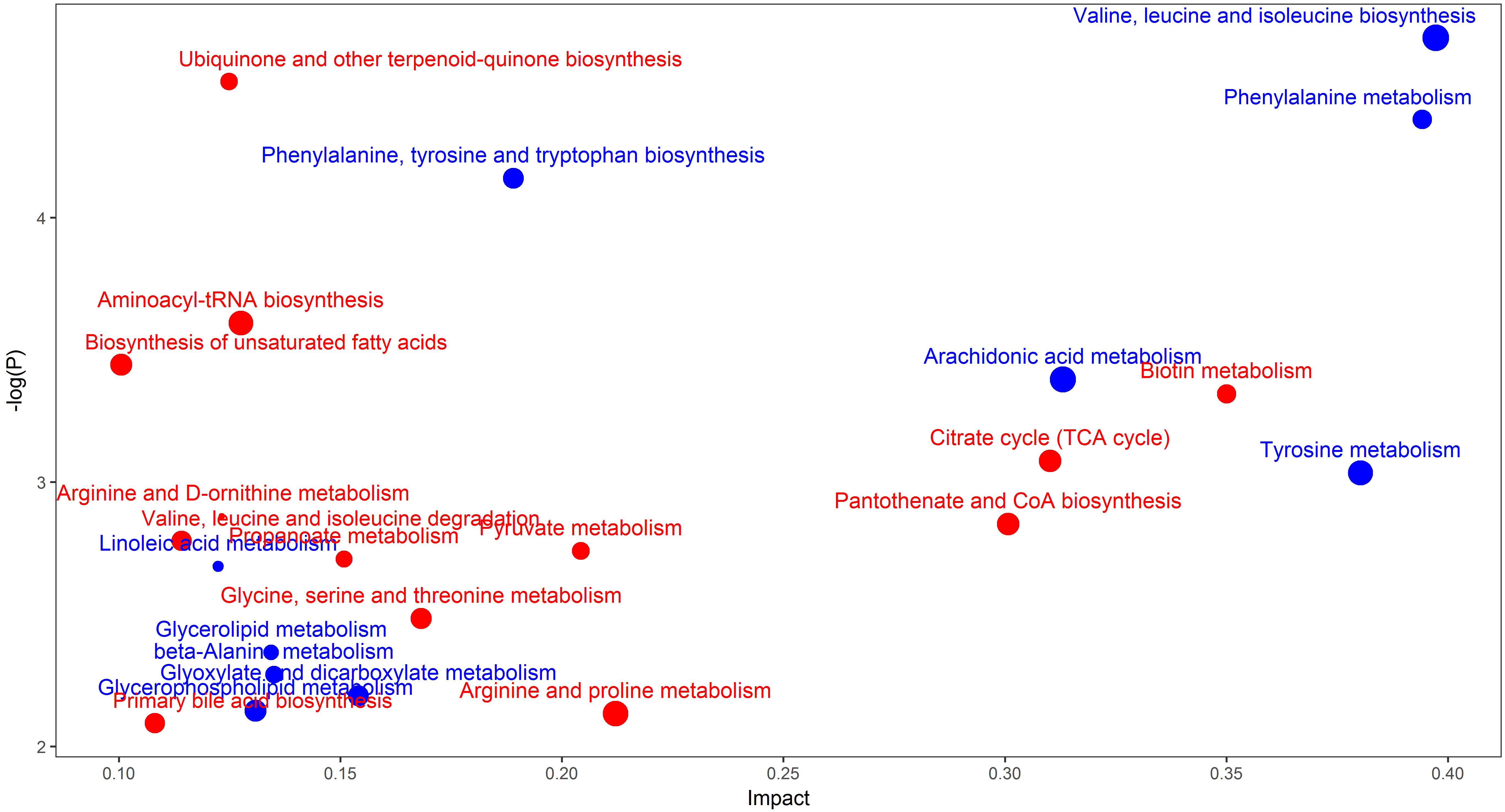


**Supplementary Figure S10. The KEGG pathways enriched by the LMP-associated serum metabolites obtained in 38 experimental pigs.** Red dots show the pathways enriched in the LMP pigs, while blue dots indicate the pathways enriched in the obese pigs (low LMP).


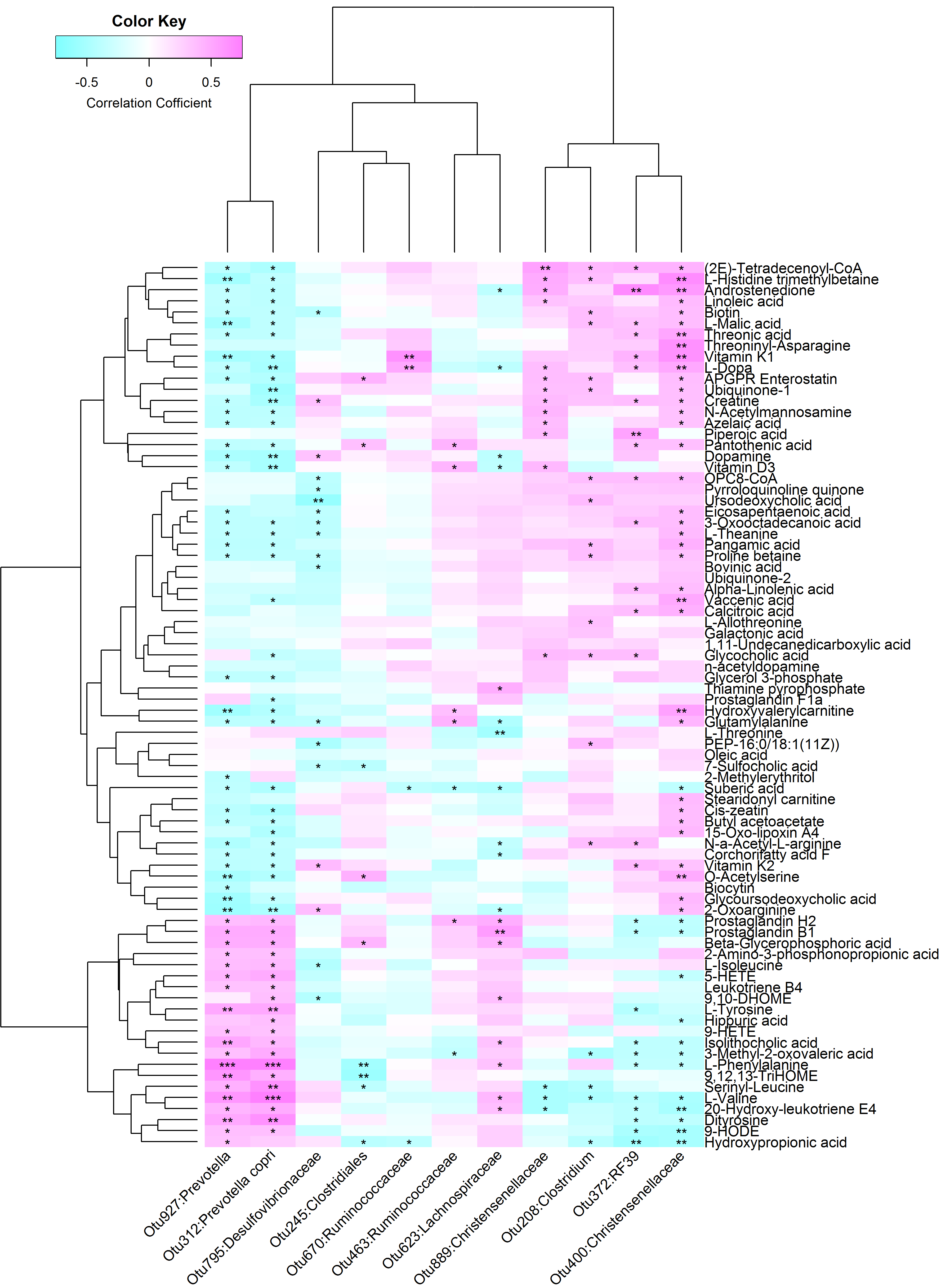


**Supplementary Figure S11. The correlation between the lean meat percentage-associated OTUs and the lean meat percentage-associated serum metabolites.** The gradient of colors shows the strength of correlation coefficient. FDRs are denoted: *, FDR < 0.05; **, FDR < 0.01, and ***, FDR < 0.005.


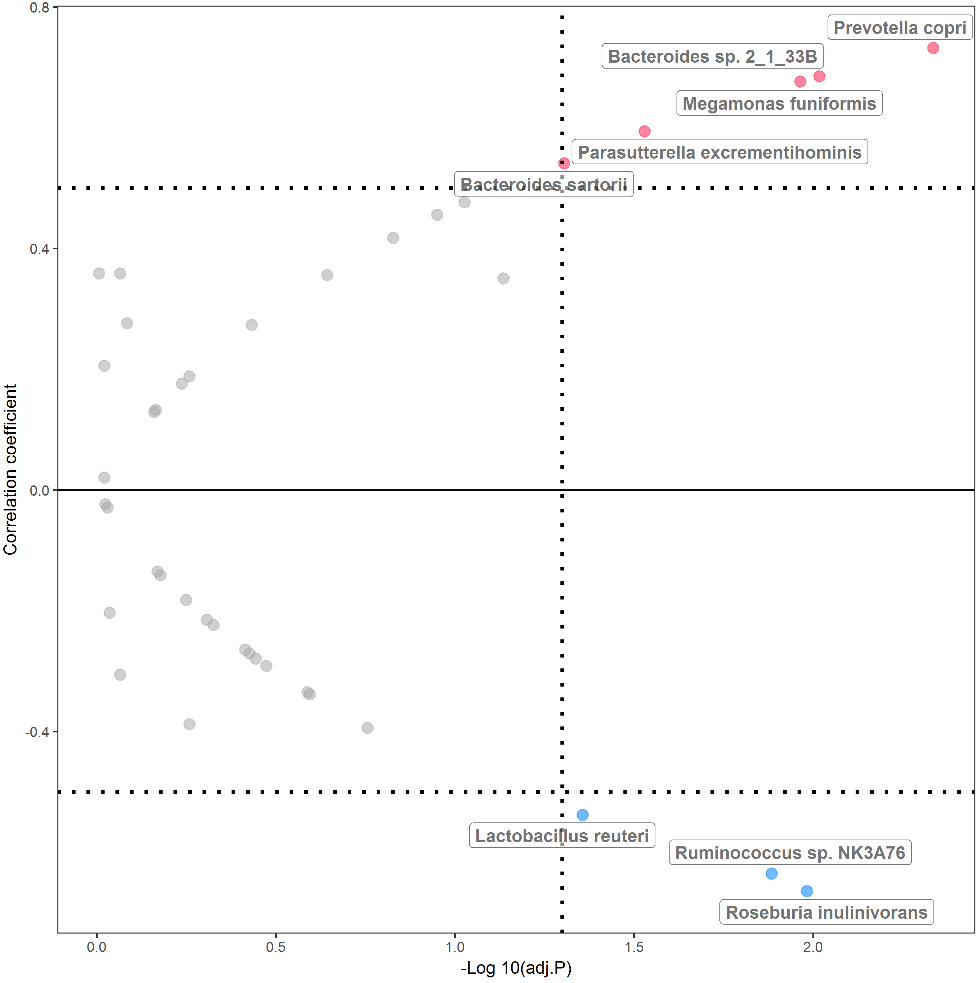


**Supplementary Figure S1****2. The bacterial species associated with the concentration of serum BCAA.** The *X*- and *Y*- axis show the P values and correlation coefficients between the abundances of bacterial species and the concentrations of serum BCAA. *P. copri* largely drove the concentration of serum BCAA.


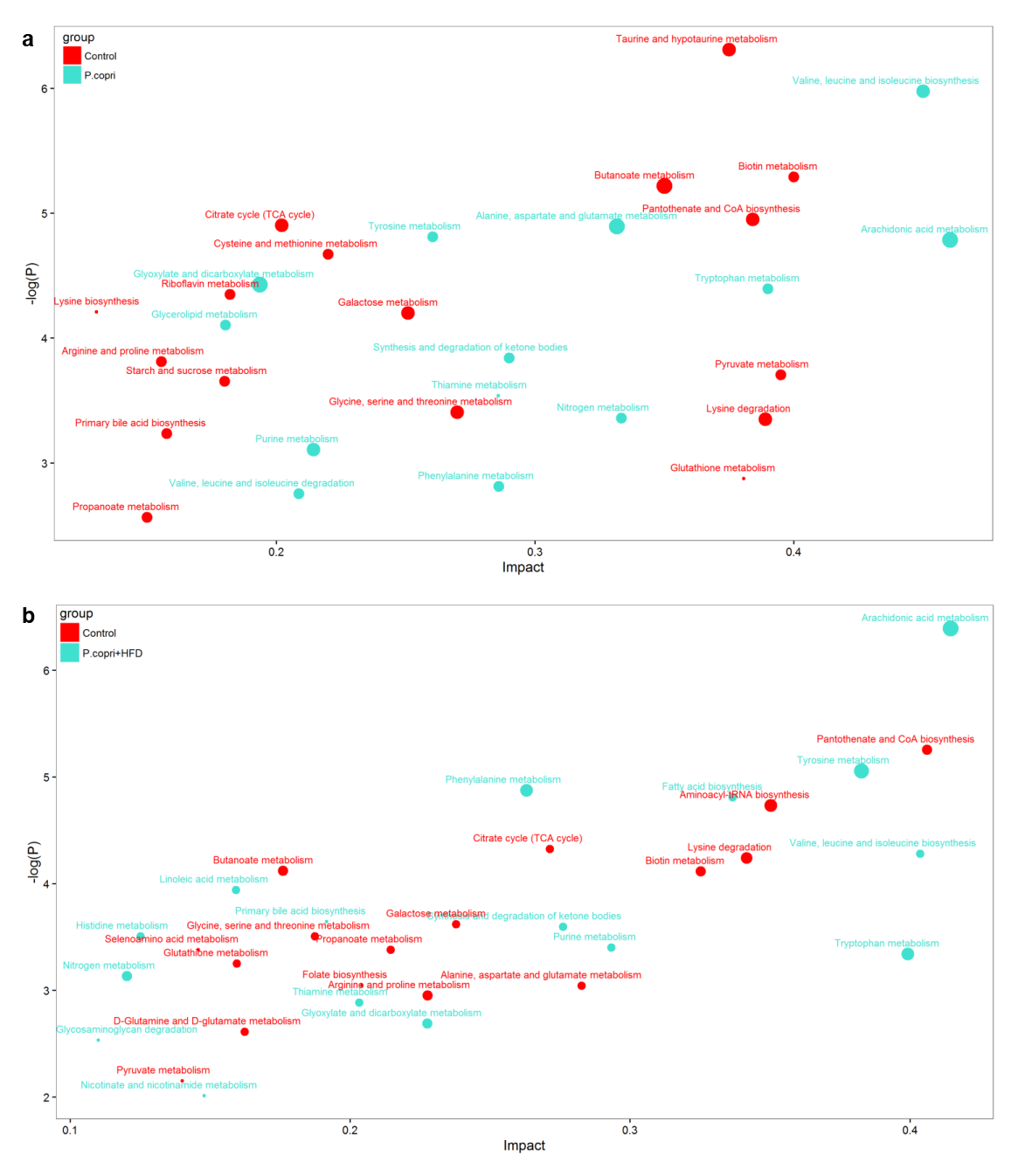


**Supplementary Figure S13.** **The KEGG pathways enriched by the differential serum metabolites between control mice and *P. copri* colonized mice (a), and between control mice and *P. copri* colonized mice fed with high fat diets (b).**


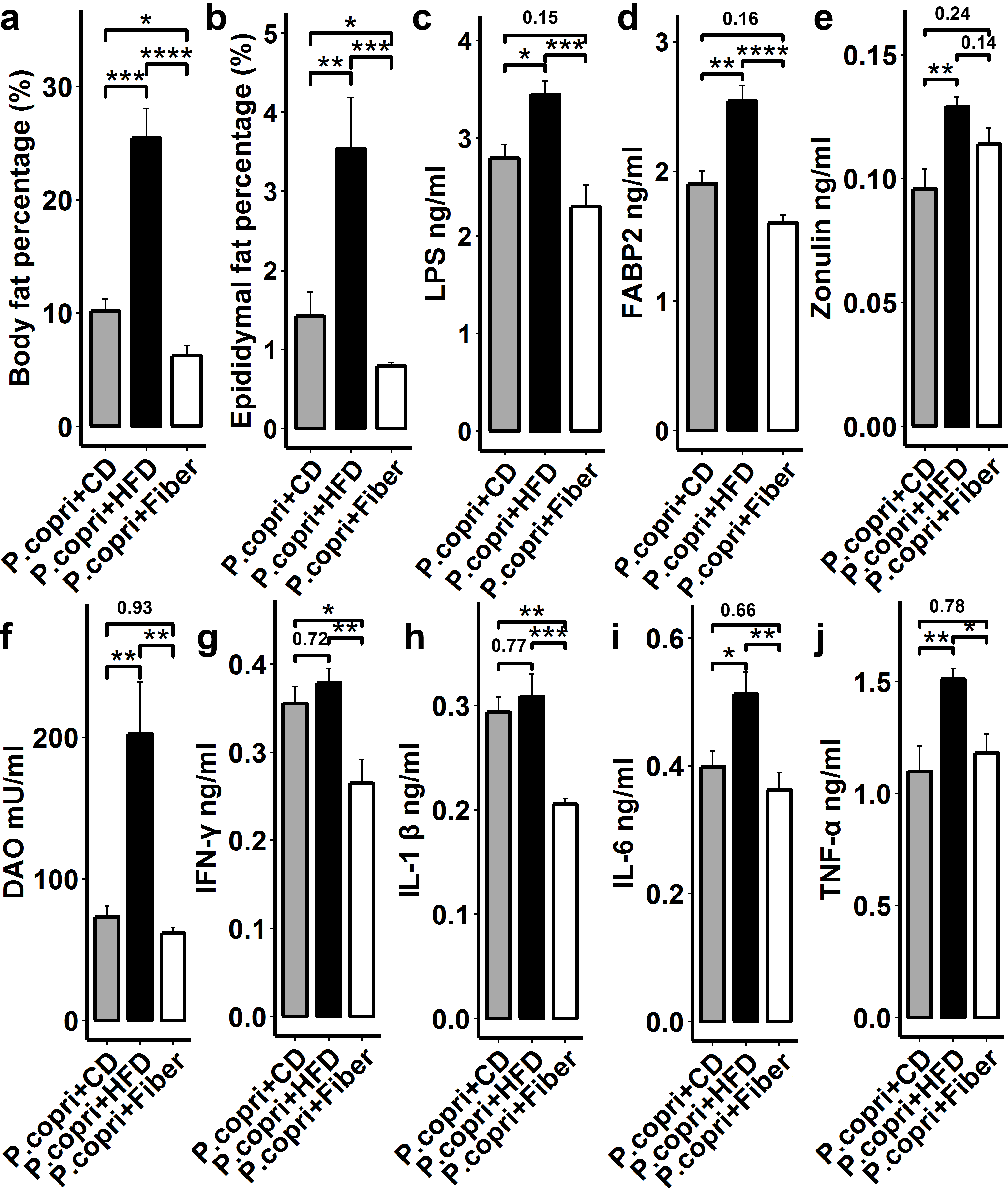


**Supplementary Figure S14. Diet effect on *P. copri* colonization and host fat accumulation with germ-free mice (C57BL6)**. The 18 germ-free mice were divided into three groups fed standard chow (n = 6), high fat diet (n = 6) and high fiber diet (n = 6). (a) qPCR confirmed the successful colonization of *P. copri* in germ-free mice and found the significant effect of diets on colonization of *P. copri*. The *Y*-axis indicates the RQ values reflecting the relative abundance of bacterial species gavage with mice. (b) Comparison of serum concentrations of LPS endotoxin among experimental mouse groups. (c) Comparison of serum concentrations of zonulin and FABP2 among experimental mouse groups. (d) Comparison of serum concentrations of pro-inflammatory cytokines (IL-1β, IL-6, TNF-α, and IFN-γ) among experimental mouse groups. *, *P* < 0.05; **, *P* < 0.01; ***, *P* < 0.005, and ****, *P* < 0.001, all P values were adjusted the multiple tests.


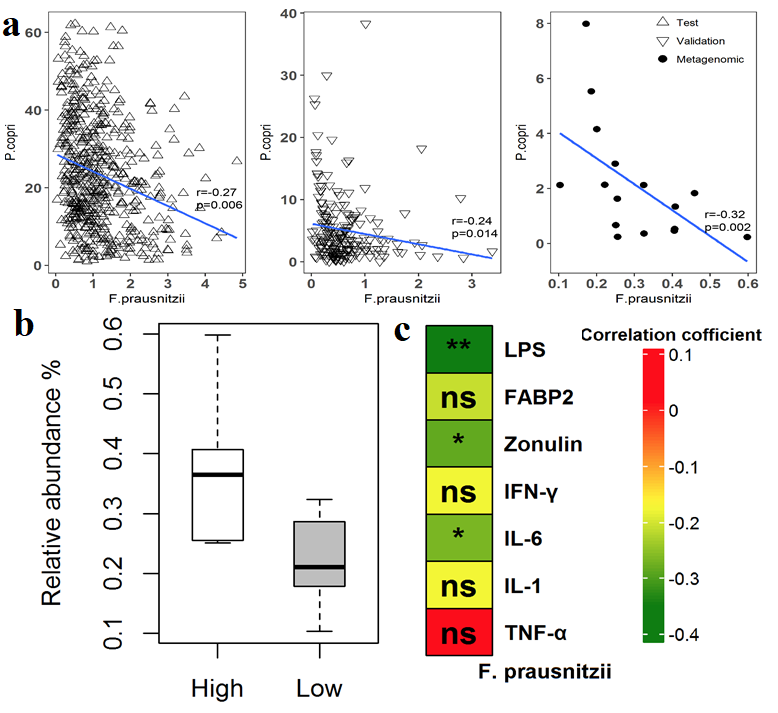


**Supplementary Figure S15. Association of *F. prausnitzii* with *P. copri*, lean meat percentage and chronic inflammation.** (a). *F. prausnitzii* was significantly associated with *P. copri* in abundance in both discovery and validation cohorts, and metagenomic sequencing data. (b). The pigs with high lean meat percentage had higher abundance of *F. prausnitzii* in the gut. (c). Association of gut *F. prausnitzii* abundance with LPS, biomarkers of intestinal barrier permeability, and pro-inflammatory cytokines.
