# Supplementary material for: Prevotella copri increases fat accumulation in pigs fed with formula diets
Source: Microbiome. 2021 Aug 21;9:175. doi: 10.1186/s40168-021-01110-0 (PMC8380364; doi:10.1186/s40168-021-01110-0)
Supplement: Supplementary file 4 — Additional file 3. [file 40168_2021_1110_MOESM3_ESM.pdf]

The availability and key parameters of software and algorithms for 16S RNA gene sequencing analysis are listed as follows:

Software/Algorithms	Parameters	Availability
FLASH (v1.2.11)	-p 33 -m 15 -M 300 -x 0.1 -z	http://www.cbcb.umd.edu/software/flash
USEARCH (v7.0.1090)	default	http://www.drive5.com/usearch
QIIME (v1.80)	-m rdp -c 0.8 --rdp_max_memory 6000	http://qiime.org/
R (v3.5.1)	statistical analysis and visualization software	https://www.r-project.org/

The availability and key parameters of software and algorithms for metagenomic sequencing analysis are listed as follows:

Software/Algorithms	Parameters	Availability
SOAPdenovo (v.2.21)	-K 55 -d 1-M 3 -F -u	http://soap.genomics.org.cn/soapdenovo
USEARCH (v7.0.1090)	default	http://www.drive5.com/usearch
MetaGeneMark (v2.10)	-a -d -f G -p 1	http://exon.gatech.edu/GeneMark/metagenome/Prediction
Cd-hit (v4.6.1)	-G 0 –aS 0.9 -c 0.95 –g 1	http://www.bioinformatics.org/cd-hit/
MOCAT (v2.0)	-sf -f -identity 95	https://mocat.embl.de
BLAST+LCA algorithm	default	http:kiwi.cs.dal.ca/Software/FCP
BLASTP	-query -db –evalue 1e-5	ftp:ftp.ncbi.nlm.nih.gov/blast/executables/blast+/LATEST/
R (v3.5.1)	statistical analysis and visualization software	https://www.r-project.org/
LEfSe	statistical analysis and visualization software	http://huttenhower.sph.harvard.edu/galaxy/
Cytoscape (v.3.6.0)	visualization software	https://cytoscape.org/

The availability and key parameters of software and algorithms for RNA-sequencing analysis are listed as follows:

Software/Algorithms	Parameters	Availability
Hisat2 (v2.1.0)	-p -x -1 -2	https://ccb.jhu.edu/software/ hisat2
Samtools (v1.8.0)	view -bS sort -o	http://www.htslib.org
StringTie (v1.3.4)	-f 0.05 -m 150 -A -b -e -G	https://ccb.jhu.edu/software/ stringTie
Ballgown (v3.5)	default	http://bioconductor.org/
R (v3.5.1)	statistical analysis and visualization software	https://www.r-project.org/

The availability and key parameters of software and algorithms for whole-genome sequencing analysis of *P. copri*

Software/Algorithms	Parameters	Availability
Canu (v1.7.11)	-correct -trim -assemble	https://github.com/marbl/canu
Pilon (v1.22)	--frags --jumps --fix --vcf	https://github.com/broadinstitute/pilon
Circlator (v1.5.5)	all --verbose	https://github.com/sanger-pathogens/circlat0r
Samtools (v1.8.0)	view -H merge -f	http://www.htslib.org
Minimap2 (v2.11-r797)	-t 30 -ax -uf	https://github.com/lh3/minimap2
Prodigal (v2.6.3)	-a -d -f gff-g 11	https://github.com/hyattpd/Prodigal
Blast2GO	default	https://www.blast2go.com

The availability and key parameters of software and algorithms for serum metabolome analysis as follows:

Software/Algorithms	Parameters	Availability
MassLynx (v 4.10.0)	default	https://micromass-masslynx.updatestar.com
MetaboAnalyst 4.0	statistical analysis and visualization software	https://www.metaboanalyst.ca
R (v3.5.1)	statistical analysis and visualization software	https://www.r-project.org/
LEfSe	statistical analysis and visualization software	http://huttenhower.sph.harvard.edu/galaxy/

Two-part model analysis

```
require(stats)
twoPart_parametric= function(rarefyOTU=NULL, trait=NULL, covariates=NULL, log2.transform=F) {
    if (is.null(rarefyOTU) | is.null(trait) ) {
        cat ("rarefyOTU\n")
        cat("trait\n")
        cat("covariates\n")
        stop()
    }
    k  =  length(rarefyOTU)
    k0 =  which(rarefyOTU == 0 )
    k1 =  which(rarefyOTU  > 0 )
    p1 =  c(0, 0, 0, 1)
    p2 =  c(0, 0, 0, 1)
    if (length(k0)>= 20 & length(k1)>=20) {
        c1  =  rep(0, length=length(rarefyOTU))
        c1[k1]  =  1
```

```
		if (is.null(covariates)) {
			model1	=	lm(trait~c1)
		} else {
			model1	=	lm(trait~covariates+c1)
		}
		if(is.element("c1", rownames(summary(model1)$coefficients))) {
			p1		=	summary(model1)$coefficients["c1",]
		}
	}
	if (length(k1)>= 20) {
		if (log2.transform) {
			c2	=	log2(rarefyOTU[k1])
		} else c2	=	rarefyOTU[k1]
		if (is.null(covariates)) {
			model2	=	lm(trait[k1]~c2)
		} else {
			model2	=	lm(trait[k1]~covariates[k1,]+c2)
		}
		if(is.element("c2", rownames(summary(model2)$coefficients))) {
			p2		=	summary(model2)$coefficients["c2",]
		}
	}
	p1.z =	abs(qnorm(p1[4]/2))
	p2.z =	abs(qnorm(p2[4]/2))
	if (!is.na(p1[3]) & p1[3]<0) p1.z = -1*p1.z
	if (!is.na(p2[3]) & p2[3]<0) p2.z	=	-1*p2.z
	w.meta.z	=	(p1.z*k+p2.z*length(k1))/sqrt(k^2+length(k1)^2)
	w.meta.p	=	(1-pnorm(abs(w.meta.z)))*2
	uw.meta.z		=	(p1.z+p2.z)/sqrt(2)
	uw.meta.p		=	(1-pnorm(abs(uw.meta.z)))*2
	minP			=	 p.adjust(min(c(p1[4], p2[4], uw.meta.p)),"fdr")
	z	=	c(p1.z, p2.z, uw.meta.z)
	asso.z	=	z[which(abs(z)==max(abs(z)))]

	result	=	data.frame(
		absentN			=	length(k0),
		presentN		=	length(k1),
		meanPresentedCounts		=	mean(rarefyOTU[k1]),
		p1.estimate		=	p1[1],
		p1.se			=	p1[2],
		p1.tvalue		=	p1[3],
		p1.pvalue		=	p1[4],
		p2.estimate		=	p2[1],
		p2.se			=	p2[2],
```

```
        p2.tvalue     =  p2[3],
        p2.pvalue     =  p2[4],
        p.combine        =  sqrt(p1[4]*p2[4]),
        w.meta.z         =  w.meta.z,
        w.meta.p         =  w.meta.p,
        uw.meta.z           =  uw.meta.z,
        uw.meta.p           =  uw.meta.p,
        asso.z   =  asso.z,
        asso.P   =  minP)

    return(result)
}
```
